# Sex‐Specific Effects of Dexmedetomidine on Brain Microvascular Endothelial Cell Function After Traumatic Brain Injury in Juvenile Mice

**DOI:** 10.1002/cns.71204

**Published:** 2026-10-12

**Authors:** Yara Mashal, Jie Fan, Tia Atoui, Zahrah Naseer, Sara Moughni, Iman Tawil, Zhi Zhang

**Affiliations:** ^1^ Department of Natural Sciences, College of Arts, Sciences, and Letters University of Michigan‐Dearborn Dearborn Michigan USA

**Keywords:** blood–brain barrier, dexmedetomidine, endothelial cells, inflammation, TBI, tight junction

## Abstract

**Aims:**

This study aimed to evaluate the effects of dexmedetomidine (DEX), a selective α2‐adrenergic receptor agonist, on brain microvascular endothelial cell (BMEC) function and blood–brain barrier (BBB) integrity in juvenile mice following traumatic brain injury (TBI). We specifically assessed the effects of DEX on ER stress, neuroinflammation, and ferroptosis and explored potential sex‐specific responses.

**Methods:**

Male and female juvenile mice were subjected to an impact‐acceleration TBI model. DEX was administered during the acute phase post‐injury. Muscle strength and sensorimotor coordination were measured, and BBB permeability was assessed. BMECs and cerebral microvessels were isolated from the injured and control regions for molecular analysis of inflammatory markers, ER stress activation, ferroptosis, and expression of tight junction markers.

**Results:**

DEX treatment improved motor and coordination outcomes and reduced BBB leakage in both sexes after TBI. Molecular analyses revealed decreased inflammatory signaling, ER stress, and ferroptosis‐related oxidative stress pathways in BMECs with DEX treatment. Importantly, DEX led to greater upregulation of selected tight junction markers in female mice than in male mice, suggesting possible sex‐specific differences in α2‐adrenergic receptor expression.

**Conclusion:**

DEX provides both behavioral and cellular protection following juvenile TBI, with potential sex‐dependent effects on BMECs and BBB function.

## Graphical abstract

Traumatic brain injury (TBI) induces acute, sex‐specific activation of endoplasmic reticulum (ER) stress, inflammatory signaling, and ferroptotic mechanisms within brain microvascular endothelial cells (BMECs). Administration of dexmedetomidine (DEX) produces both sex‐dependent and sex‐independent modulatory effects on BMECs, prominently suppressing inflammatory cascades and diminishing ferroptosis‐associated markers across both male and female cohorts. However, the extent and qualitative nature of these DEX‐mediated responses display distinct sex‐related divergences, underscoring differential regulatory dynamics in BMEC functional recovery post‐TBI.

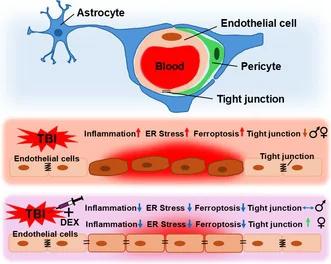

## 1 Introduction

Traumatic brain injury (TBI) remains a major global health concern [1]. Despite significant advances in understanding the complex pathophysiology of TBI, effective treatment options remain limited. Key pathophysiological features of TBI include cerebrovascular dysfunction and disruption of the blood–brain barrier (BBB) [2, 3]. Both processes contribute to secondary injury mechanisms, including endoplasmic reticulum (ER) stress and neuroinflammation, that promote neuronal damage and cell death [4, 5]. Moreover, BBB breakdown and microvascular injury following TBI facilitate iron accumulation in the brain parenchyma, promoting ferroptosis and associated neuronal membrane damage [6].

The BBB is primarily composed of brain microvascular endothelial cells (BMECs), pericytes, and astrocytes, which together form an integral part of the neurovascular unit [7]. The integrity and selective permeability of the BBB are maintained by tight junctions (TJs), specialized transporters, and efflux systems within BMECs [8]. At the molecular level, endothelial TJs consist of claudins, occludin, and junctional adhesion molecules (JAMs), reinforced by accessory proteins such as zonula occludens that tether TJs to the actin cytoskeleton [9]. Claudins and occludin govern paracellular transport, while JAMs facilitate cell adhesion and leukocyte transmigration [10]. Following TBI, activated glial cells release mediators such as reactive oxygen species (ROS), which disrupt tight junctions and compromise BBB integrity [11, 12]. BMEC dysfunction and increased BBB permeability develop within hours after TBI, promoting cerebral edema and secondary injury [13]. Few interventions have been shown to preserve or restore BMEC function after TBI.

Dexmedetomidine (DEX), a highly selective α2‐adrenergic receptor (α2‐AR) agonist, has been approved by the Food and Drug Administration (FDA) for short‐term sedation and analgesia in clinical practice, including children [14]. DEX has demonstrated neuroprotective effects in preclinical models of TBI [15] and potential benefits in patients with TBI [16]. Mechanistically, DEX suppresses neuroinflammation by modulating TLR4/NF‐κB and ROS/Nrf2 signaling [17, 18, 19]. It also mitigates ER stress and cerebral edema through the PERK/ATF4/CHOP pathway [20] and limits neuronal oxidative damage through modulation of PPARγ/PGC‐1α signaling [21]. DEX has also been shown to preserve endothelial integrity and BBB function in stroke and sepsis models [22, 23, 24]. Notably, the immature cerebral vasculature is more fragile and susceptible to injury than the adult cerebral vasculature, partly because of differential expression of transporters and junctional proteins [25]. However, the effects of DEX on BMECs after TBI in the immature brain remain underexplored. In this study, we investigated the effects of DEX on BMEC function and BBB integrity following TBI in juvenile male and female mice. We specifically examined the effects of DEX on ER stress, inflammatory responses, and ferroptosis‐related pathways in BMECs. Our goal was to evaluate the therapeutic potential of DEX and identify possible sex‐specific effects in a juvenile model relevant to pediatric TBI.

## 2 Materials and Methods

### 2.1 Animals

Male and female C57BL/6 breeder mice, aged 2–3 months, were obtained from The Jackson Laboratory (Bar Harbor, ME, USA) and maintained for in‐house breeding. Offspring generated from these breeding pairs were used in the subsequent experimental procedures. All pups were delivered naturally and remained with their dam after birth until weaning. All animals were housed under ambient conditions with free access to food and water. All experiments were conducted in accordance with the Guide for the Care and Use of Laboratory Animals, 8th edition (National Research Council, National Academies Press, 2011), and complied with the ARRIVE (Animal Research: Reporting of In Vivo Experiments) guidelines. Experimental procedures were approved by the Institutional Animal Care and Use Committee (IACUC) of the University of Michigan (Protocol number PRO00012565).

### 2.2 Estimation of Animal Numbers

Statistical power and sample size calculations were performed using “PS: power and sample size calculation” software (version 3.1.6; Department of Biostatistics, Vanderbilt University, USA). Power calculations were performed with a significance level (*α*) of 0.05 (two‐tailed), power of the study (1 − *β*) of 80%, and variances (*δ*, *σ*, and *m*) estimated from our preliminary data. Based on these parameters, the required sample sizes were estimated to be 5–10 animals per group for behavioral assessments and 4–6 animals per group for immunohistochemical and quantitative real‐time PCR (qPCR) analyses. In addition, a prespecified interim analysis was performed in accordance with the principles of replacement, reduction, and refinement to avoid unnecessary use of experimental animals.

### 2.3 Impact‐Acceleration Model of TBI

On postnatal days 20–21 (P20‐21), male (M) and female (F) animals (*n* = 128, 64M/64F) from the same breeding cohort were randomized into Sham (*n* = 44, 22M/22F), TBI + saline (*n* = 44, 22M/22F), and TBI + DEX (*n* = 40, 20M/20F) groups using a random number generator. Randomization was stratified by sex. Animals in the TBI groups underwent injury procedures as previously described [26, 27, 28, 29, 30, 31] (see Data S1 for detailed materials and methods). Sham animals were anesthetized with 4% isoflurane without TBI impact.

### 2.4 Enzymatic Isolation of Primary BMECs and Ferroptotic Lipid Peroxidation Evaluation

Animals (Sham = 4 [2M/2F]; TBI = 4 [2M/2F]) were euthanized and brains were harvested. The cortical injury regions (approximately between bregma +2 mm and bregma −1 mm) in the TBI mice and corresponding regions in the sham mice were microdissected as previously described [27]. Primary BMECs were isolated according to a previously published protocol [32]. Cell purity was calculated as follows: Cell purity (%) = (number of CD31‐positive cells/total number of cells) × 100 [29, 30]. Using this method, cell purity was determined to be 98% ± 2%. To investigate the impact of TBI on ferroptosis, BMECs (320 cells/mm^2^) were plated and cultured in the micro‐8‐well slide (ibidi USA Inc., WI, USA). Cells were then incubated with BioTracker 633 Red Mitochondria Dye (100 nM; MilliporeSigma, MA, USA) and BioTracker Mitochondrial Lipid Peroxide Live Cell Ferroptosis Indicator (100 nM; MilliporeSigma, MA, USA) for 30 min before imaging. Images were acquired using a Nikon Plan Apo 20×/0.9 objective mounted on a Nikon Eclipse TS2R fluorescence microscope (Nikon, NY, USA).

### 2.5 Drug Administration

Dexdomitor (0.5 mg/mL; MWI Animal Health, Fenton, MI, USA) was diluted in 0.9% sterile saline to a concentration of 10 μg/mL. Animals in the TBI + DEX group received an intraperitoneal (IP) injection of DEX (25 μg/kg) at 15 min post‐injury [30]. The TBI + saline group received the same volume of saline at 15 min post‐injury. The sham group did not receive any treatment.

### 2.6 Body Weight Measurement and Behavioral Testing

Mice from the Sham (*n* = 40, 20M/20F), TBI + saline (*n* = 40, 20M/20F), and TBI + DEX (*n* = 40, 20M/20F) groups underwent body weight measurement and behavioral testing before injury (baseline) and at 6‐h post‐injury. The personnel were blinded to experimental groups. Changes in body weight were calculated as (body weight)_change_ = (body weight)_6h_ − (body weight)_baseline_. Muscle strength was evaluated with a grip strength test using a grip strength meter (BIOSEB, FL, USA) as previously published [29, 30]. The change in the grip strength before and after injury was calculated as: (grip strength)_change_ = (grip strength)_6h_ − (grip strength)_baseline_. Sensorimotor coordination, endurance, and fatigue resistance were evaluated with a touchscreen five‐station accelerating Panlab Rotarod for mice (BIOSEB, FL, USA) as previously published [29, 30, 33]. The change in the latency to the first fall before and after injury was calculated as: (latency)_change_ = (latency)_6h_ − (latency)_baseline_.

### 2.7 Evans Blue Extravasation Assay

Evans blue extravasation assay was modified based on published protocols [34, 35]. Animals (Sham = 10 [5M/5F]; TBI + saline = 10 [5M/5F]; TBI + DEX = 10 [5M/5F]) received an intravenous injection of 10 mg/kg of 2% sterile Evans blue (Thermo Fisher Scientific, MA, USA) via the tail vein after completion of behavioral tests. Animals were euthanized at 2 h postinjection. Brains were removed, and the cortical injury regions were microdissected [27, 31, 36]. Brain tissues were weighed, homogenized in a 50% solution of trichloroacetic acid (Sigma‐Aldrich, MA, USA), and centrifuged at 10,000 × *g* for 20 min. The supernatant absorbance was measured at 610 nm using a NanoDrop 2000 Spectrophotometer (Thermo Fisher Scientific, MA, USA). Evans blue absorbance was quantified according to an Evans blue external standard curve (1–50 ng/mL) and converted into nanograms of dye extravasated per milligram of tissue. The Evans blue concentrations were then normalized to the values obtained from the sham animals and compared among treatment groups.

### 2.8 Isolation of Cerebral Microvessels and Immunohistochemistry

Animals were euthanized after completion of behavioral tests (Sham = 8 [4M/4F]; TBI + saline = 8 [4M/4F]; TBI + DEX = 8 [4M/4F]). The cortical injury regions were microdissected as previously described [27, 31, 36]. The cerebral microvessels were isolated based on a published protocol [37]. The isolated microvessels were incubated with primary antibodies overnight at 4°C. The primary antibodies used in this study were as follows: rat anti‐CD31 (an endothelial marker; 1:250; Thermo Fisher Scientific, MA, USA), goat anti‐ADRA2A (1:50; Thermo Fisher Scientific, MA, USA), rabbit anti‐ADRA2B (1:50; Thermo Fisher Scientific, MA, USA), rabbit anti‐Junctional Adhesion Molecule 1/JAM‐A (1:250; Abcam, MA, USA), rabbit anti‐occludin (1:100; Abcam, MA, USA), mouse anti‐VE‐cadherin (1:100; Santa Cruz Biotechnology, TX, USA), mouse anti‐TNF‐α (1:250; GeneTex, CA, USA), rabbit anti‐ICAM‐1 (1:50; Thermo Fisher Scientific, MA, USA), rabbit anti‐phospho‐IRE1α (1:250; Thermo Fisher Scientific, MA, USA), rabbit anti‐GPX4 (1:50; Thermo Fisher Scientific, MA, USA), mouse anti‐4‐HNE (1:250; Thermo Fisher Scientific, MA, USA), and mouse anti‐malondialdehyde (MDA) (1:250; Thermo Fisher Scientific, MA, USA). The microvessels were subsequently washed and incubated with fluorescent secondary antibodies (1:250; Life Technologies, MA, USA) for 2 h at room temperature. The microvessels were mounted with fluorescence mounting medium with DAPI (Sigma‐Aldrich, MO, USA). Images (40× magnification, 5 per animal) were acquired using the Nikon Eclipse TS2R fluorescence microscope (Nikon, NY, USA) with NIS‐Elements AR 5.42.04 software package, and the camera settings were kept the same for all the experimental groups. All slides and images were coded, and the analysis was performed with personnel blinded to group allocation. The measurement data were averaged per animal and compared among groups.

### 2.9 Isolation of Primary BMECs Using Magnetic Cell Separation

Animals were euthanized following completion of behavioral assessments (Sham = 12 [6M/6F]; TBI + saline = 12 [6M/6F]; TBI + DEX = 12 [6M/6F]). Brains were harvested and the cortical injury regions were microdissected [27]. Brain tissues were homogenized in ice‐cold HBSS using a Dounce tissue homogenizer and passed through 40‐μm cell strainers to obtain a single‐cell suspension. Magnetic cell separation was performed with mouse CD31 MicroBeads (catalog no. 130‐097‐418; Miltenyi Biotec, MD, USA) and a QuadroMACS Separator (Miltenyi Biotec, MD, USA) according to the manufacturer's instructions.

### 2.10 RNA Isolation and Quantitative Real‐Time Polymerase Chain Reaction (qPCR)

Total RNA was extracted using TRI Reagent (Sigma‐Aldrich, MO, USA) according to the manufacturer's instructions. RNA samples were quantified using the NanoDrop ND‐2000 Spectrophotometer (Thermo Fisher Scientific, MA, USA). Single‐stranded complementary DNA (cDNA) was reverse transcribed from RNA using the High‐Capacity cDNA Reverse Transcription Kit with RNase inhibitor (Thermo Fisher Scientific, MA, USA). qPCR was performed with iTaq Universal SYBR Green Supermix (Bio‐Rad, CA, USA) and the CFX Connect real‐time PCR detection system (Bio‐Rad, CA, USA). Amplification conditions included 30 s at 95°C, 40 cycles at 95°C for 5 s, and 60°C for 30 s. Primers were custom‐designed (Table 1) and ordered from Integrated DNA Technologies (Coralville, IA, USA). The comparative threshold cycle (Ct) method was used to assess differential gene expression. The sham group was the reference group, and glyceraldehyde 3‐phosphate dehydrogenase (GAPDH) was the housekeeping gene.

**TABLE 1:** Primer sequences used for qPCR.

Gene	Forward primer	Reverse primer
*Adra2a*	CCTGCCTGAGTGCTTAGAAA	GGGTGCTAGCCAAGAAAGAA
*Adra2b*	GAAGAGGAGGTGGAAGAATGTG	CTGCAAGGGTGGGTTGAATA
*Tnf‐α*	TCAGCCGATTTGCTATCTCATA	AGTACTTGGGCAGATTGACCTC
*Il1b*	GGTGTGTGACGTTCCCATTA	ATTGAGGTGGAGAGCTTTCAG
*Il6*	GTCTGTAGCTCATTCTGCTCTG	GAAGGCAACTGGATGGAAGT
*Tgfb1*	GGTGGTATACTGAGACACCTTG	CCCAAGGAAAGGTAGGTGATAG
*Tlr4*	GGGTATTTGACACCCTCCATAG	CAAGAGTGCTGAGGGAATACAG
*Nlrp3*	ACGTGTTCCAGAAGGAAGTG	GCCTCCTCTTCCAGCAAATA
*Nfκb*	AGACATCCTTCCGCAAACTC	TAGGTCCTTCCTGCCCATAA
*Icam1*	CCAGTACTGCTGGTCATTGT	TCCTCCTGAGCCTTCTGTAA
*Ire1a*	CTGTGGTCAAGATGGACTGG	GAAGCGGGAAGTGAAGTAGC
*Perk*	TCAAGTTTCCTCTACTGTTCACTCA	CGGGAAACTCCAAGTTCTCA
*Gpx4*	CCCACTGTGGAAATGGATGA	ACGCAGCCGTTCTTATCAA
*Ptgs2*	GAAGATTCCCTCCGGTGTTT	CCCTTCTCACTGGCTTATGTAG
*Hmox1*	GTACACATCCAAGCCGAGAA	TGGTACAAGGAAGCCATCAC
*Tfrc*	TCCTGTCGCCCTATGTATCT	CGAAGCTTCAAGTTCTCCACTA
*Inos*	GGAATCTTGGAGCGAGTTGT	CCTCTTGTCTTTGACCCAGTAG
*Nos3*	TGTGGGAGAAGATGCCAAAG	GAGTCAGCCCTGGTAGTAATTG
*Ocln*	GAGCTTACAGGCAGAACTAGAC	CAGCCATGTACTCTTCACTCTC
*Cldn5*	GCAGAGGCACCAGAATCAAT	TGGCTAAAGACTGAATGCTCAC
*Cfl1*	CCCATGCTGCCAACTTCTA	GGGAGGGTCATTTCCACATT
*Pfn1*	TCACCATGACTGCCAAGAC	CAGGTGAGAGGCCATTTCATA
*Pecam1*	CAACAGAGCCAGCAGTATGA	TGACAACCACCGCAATGA
*Zic3*	CCTCCTAGCTACTTGCTCTTTC	AGATGGACCTGGTTGTTGTC
*Gapdh*	AACAGCAACTCCCACTCTTC	CCTGTTGCTGTAGCCGTATT

### 2.11 Mouse Brain Tissue Immunofluorescence Staining and Imaging

Animals were euthanized after completion of behavioral tests (Sham = 10 [5M/5F]; TBI + saline = 10 [5M/5F]; TBI + DEX = 10 [5M/5F]). Brains were removed, postfixed in 10% formalin for 48 h, and then cryoprotected in 30% sucrose (in PBS). Coronal sections (20 μm, 1:6 series) were prepared on a cryostat (Leica Microsystems, IL, USA). Brain sections were incubated with rat anti‐CD31 (1:250; Thermo Fisher Scientific, MA, USA) and mouse anti‐CHOP (1:250; Thermo Fisher Scientific, MA, USA) overnight at 4°C. Brain sections were subsequently washed and incubated with fluorescent secondary antibodies (1:250; Life Technologies, MA, USA) for 2 h at room temperature. The slides were dried and coverslipped with fluorescent mounting medium with DAPI (Sigma‐Aldrich, MO, USA). Images (40× or 60× magnification, 5 per animal) were randomly acquired from the cortical injury region (approximately between bregma +1 mm and bregma −0.5 mm) in TBI animals. Corresponding cortical regions were imaged in sham animals. All images were acquired using a Nikon Eclipse TS2R fluorescence microscope, with identical camera settings across experimental groups. All slides and images were coded, and the analysis was performed with personnel blinded to group allocation. The measurement data were averaged per animal and compared among groups.

### 2.12 Histology Quantification

The expression of CHOP was evaluated as the “percentage of area” using the “particle analysis” function in Fiji ImageJ software (National Institutes of Health, NIH) as previously described [26, 27, 38, 39]. Co‐localization analyses of CD31^+^/CHOP^+^ in brain tissues, CD31^+^/ADRA2A^+^, CD31^+^/ADRA2B^+^, CD31^+^/JAM‐A^+^, CD31^+^/Occludin^+^, CD31^+^/VE‐cadherin^+^, CD31^+^/TNF‐α^+^, CD31^+^/ICAM‐1^+^, CD31^+^/phospho‐IRE1α^+^, CD31^+^/GPX4^+^, CD31^+^/4‐HNE^+^, and CD31^+^/MDA^+^ in isolated cerebral microvessels were performed using the “Coloc 2” function in Fiji ImageJ (National Institutes of Health, NIH) following the manufacturer's instructions [29]. The co‐localization was analyzed using Kendall's tau rank correlation test, and Costes' randomization was set at 10. To ensure the accuracy of the analysis, data were manually checked with co‐localization analysis as described previously [26, 27, 30, 38, 39].

### 2.13 Statistical Analysis

Data were analyzed using GraphPad Prism (version 10.4.1; CA, USA) and IBM SPSS Statistics (version: 28.0.1.1 [15]). Data are presented as the mean ± SEM. The D'Agostino‐Pearson omnibus normality test or Kolmogorov–Smirnov test was used for normality measurement. Student's *t*‐test (two‐tailed) or Mann–Whitney *U* test (two‐tailed) was used for two‐group comparisons. Multiple comparisons were analyzed using one‐way or two‐way analysis of variance (ANOVA), followed by Bonferroni post hoc correction where appropriate. The Bonferroni method was used to adjust individual *p* values for multiple hypothesis testing and to control the family‐wise error rate. In addition, the two‐stage step‐up procedure of Benjamini, Krieger, and Yekutieli was applied, where appropriate, to control the false discovery rate. To enhance data transparency, individual data points from the imaging analyses are presented in Figure S1. Statistical significance was set at *p* < 0.05 for all analyses.

## 3 Results

### 3.1 TBI Increased Cellular Oxidative Stress and Dysregulated α2‐Adrenergic Receptor (α2‐AR) Expression in BMECs

Mitochondrial lipid peroxide levels were elevated in BMECs from both male and female TBI groups versus Sham (Figure 1A), indicating increased lipid peroxidation consistent with ferroptosis‐related oxidative stress and cellular dysfunction. Because DEX is a highly selective α2‐AR agonist, we evaluated the effects of TBI on the expression of α2A‐adrenergic receptor (α_2A_‐AR) and α2B‐adrenergic receptor (α_2B_‐AR) in the primary BMECs. We found that the mRNA levels of *Adra2a* (*F*_1,20_ = 15.96, *p* = 0.0007) and *Adra2b* (*F*_1,20_ = 9.897, *p* = 0.0051) were significantly decreased in the female TBI (*Adra2a*, *p* = 0.0078; *Adra2b*, *p* = 0.0066) compared with the Sham (Figure 1C,D). Moreover, *Adra2b* mRNA expression was significantly decreased in the primary BMECs isolated from the female TBI compared with the male TBI (*p* = 0.0432) (Figure 1D). In isolated cerebral microvessels, BMEC α_2A_‐AR and α_2B_‐AR protein levels were significantly reduced in female TBI animals compared with female Sham and male TBI animals. α_2A_‐AR showed a significant treatment effect (*F*_1,12_ = 14.13, *p* = 0.0027), while α_2B_‐AR showed significant treatment (*F*_1,12_ = 11.24, *p* = 0.0058) and treatment × sex interaction effects (*F*_1,12_ = 4.883, *p* = 0.0473) (Figure 1B,E,F). The increased lipid peroxidation and potential sex‐specific changes in α2‐AR expression in BMECs underscore the need to investigate the sex‐specific responses to DEX treatment during the acute phase after TBI.

**FIGURE 1:**
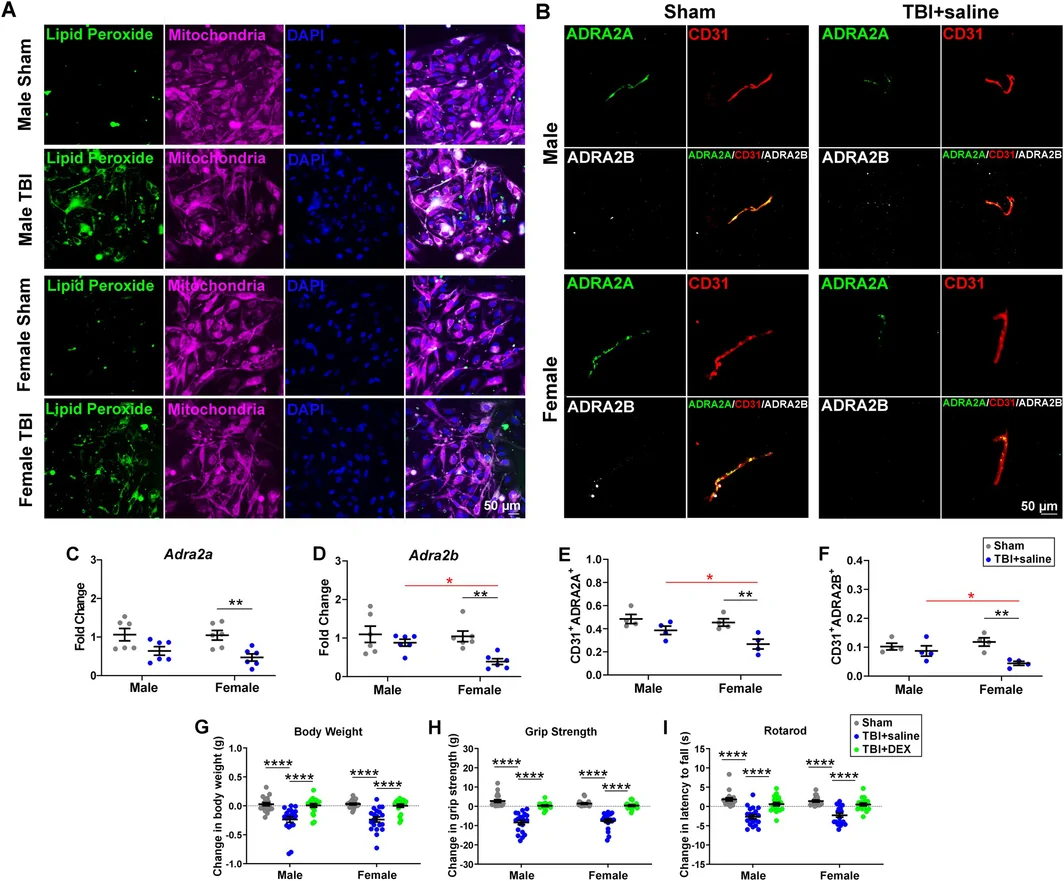
Dysregulation of BMECs and the effects of DEX on body weight and sensorimotor functions. (A) Representative images from male and female Sham and TBI groups illustrate increased co‐localization of lipid peroxides (oxidative stress marker; green) with mitochondria (magenta) in isolated BMECs of both male and female TBI mice compared with Sham controls. Nuclear staining was performed using DAPI (4′,6‐diamidino‐2‐phenylindole; blue). Scale bar: 50 μm. (B) Representative images of isolated blood vessels from male and female Sham and TBI groups showing immunostaining for α_2A_‐AR (green), α_2B_‐AR (white), and the BMEC marker CD31 (red). Scale bar: 50 μm. (C) mRNA expression of *Adra2a* was significantly decreased in the isolated BMECs from the female TBI group (*n* = 6 per group). (D) mRNA expression of *Adra2b* was significantly decreased in the isolated BMECs from the female TBI group compared with the female Sham and male TBI groups (*n* = 6 per group). (E) α_2A_‐AR protein expression in BMECs was significantly reduced in blood vessels isolated from the female TBI group (*n* = 4 per group). (F) α_2B_‐AR protein expression in BMECs was significantly reduced in blood vessels isolated from the female TBI group (*n* = 4 per group). (G) Body weight gain was significantly reduced in the male and female TBI + saline groups compared with both male and female Sham and TBI + DEX groups (*n* = 20 per group). (H) Grip strength was significantly decreased in both male and female TBI + saline groups compared with the male and female Sham and TBI + DEX groups (*n* = 20 per group). (I) In the rotarod test, the latency to first fall was significantly decreased in both male and female TBI + saline groups, compared with the male and female Sham and TBI + DEX groups (*n* = 20 per group). Statistical analysis was performed using two‐way ANOVA (sex [male, female], treatment [Sham, TBI + saline, TBI + DEX]) and Bonferroni's multiple‐comparison test. *p < 0.05; **p < 0.01; ****p < 0.0001. Red annotations indicate sex‐related effects, whereas black annotations indicate treatment‐related effects.

### 3.2 DEX Attenuated TBI‐Induced Reductions in Body‐Weight Gain, Muscle Strength, and Sensorimotor Coordination

The body weight gain (*F*_2,114_ = 35.73, *p* < 0.0001), grip strength (*F*_2,114_ = 115.0, *p* < 0.0001), and the latency to the first fall (*F*_2,114_ = 50.58, *p* < 0.0001) were significantly decreased in the TBI + saline groups compared with the Sham and TBI + DEX groups in both males and females based on treatment (Figure 1G–I).

### 3.3 DEX Improved BBB Integrity

Evans blue levels were significantly higher in both male and female TBI + saline and TBI + DEX groups compared with the Sham based on treatment (*F*_2,24_ = 47.02, *p* < 0.0001). Moreover, the Evans blue level was significantly higher in the male and female TBI + saline groups compared with the TBI + DEX (Figure 2A,B). Thus, these findings support partial attenuation of TBI‐induced BBB disruption by DEX rather than complete restoration of BBB integrity.

**FIGURE 2:**
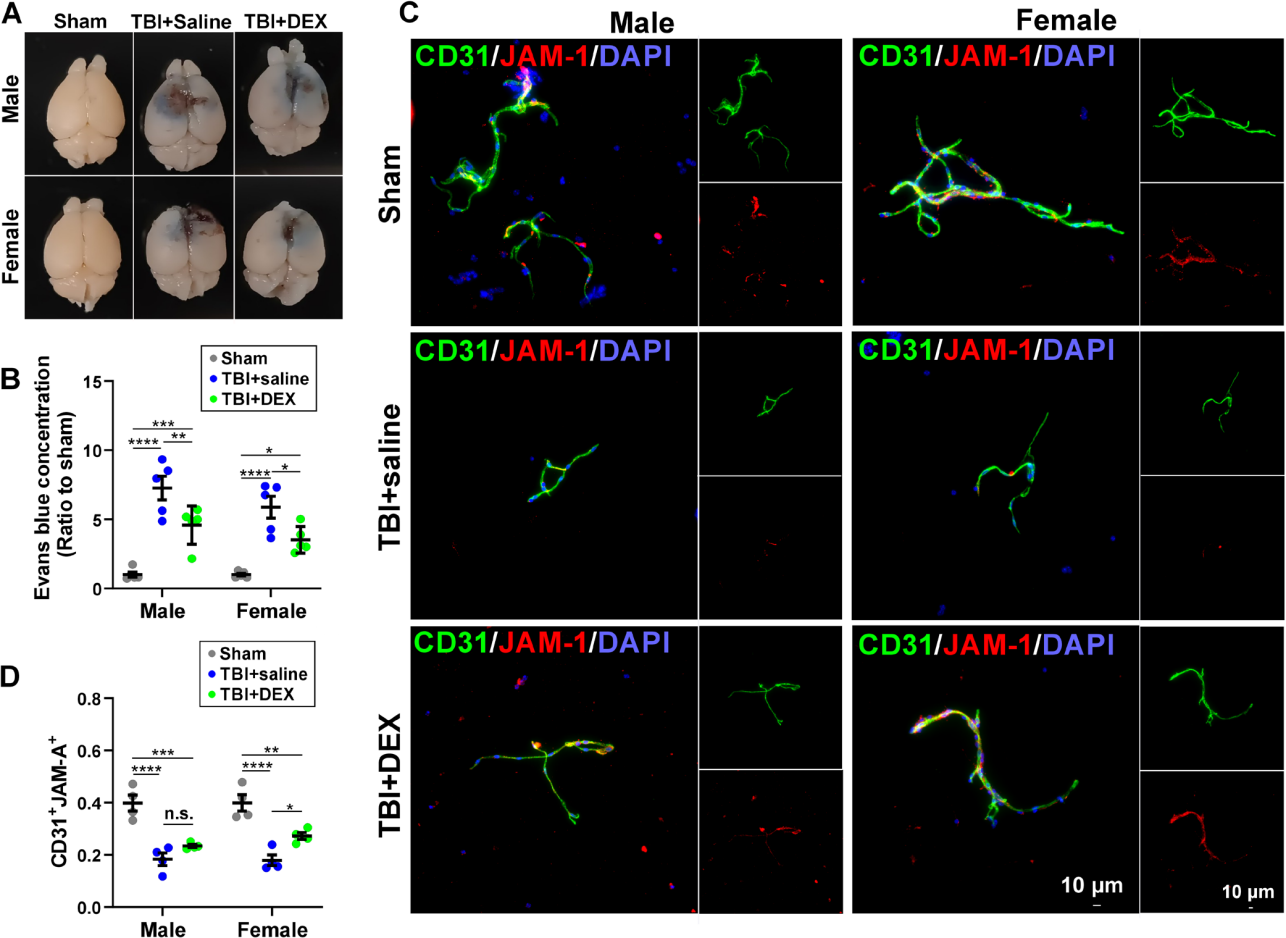
Extravasation of Evans blue and tight junction protein evaluation. (A) Representative images of brains from Sham, TBI + saline, and TBI + DEX groups injected with Evans blue are shown for both males and females. (B) Evans blue concentrations were significantly elevated in tissue samples from injured brain regions in both male and female TBI + saline and TBI + DEX groups compared with Sham controls. Furthermore, Evans blue concentration was markedly higher in the TBI + saline group than in the TBI + DEX group (*n* = 5 per group). (C) Representative images showing co‐localization of JAM‐A with the BMEC marker CD31 in isolated microvessels from Sham, TBI + saline and TBI + DEX groups in both males and females. CD31 (green; a BMEC marker); JAM‐A (red; a junctional adhesion protein); DAPI (blue; a nuclear marker). Scale bar: 10 μm. (D) Quantitative analysis of CD31 and JAM‐A co‐localization on isolated cerebral blood vessels (*n* = 4 per group). Statistical analysis was performed using two‐way ANOVA (sex [male, female], treatment [Sham, TBI + saline, TBI + DEX]), and Bonferroni's multiple‐comparison test. *p < 0.05; **p < 0.01; ***p < 0.001; ****p < 0.0001; n.s., not significant.

The expression of JAM‐A in endothelial cells (CD31^+^JAM‐A^+^) in the isolated blood vessels was significantly lower in both male and female TBI + saline and TBI + DEX groups compared with the Sham based on treatment (*F*_2,18_ = 46.93, *p* < 0.0001). Moreover, the expression of JAM‐A in the endothelial cells (CD31^+^JAM‐A^+^) was significantly lower in the female TBI + saline compared with the female TBI + DEX (Figure 2C,D).

### 3.4 Sex‐Specific Effects of DEX on Tight Junction Markers in BMECs

To assess the impact of DEX on BMEC function, the mRNA levels of *Inos*, *Nos3*, *Ocln*, *Cldn5*, *Cfl1*, *Pfn1*, *Pecam1*, and *Zic3* were measured in BMECs isolated from both male and female mice. *Inos* (*F*_2,30_ = 11.7, *p* = 0.0002), *Nos3* (*F*_2,30_ = 13.68, *p* < 0.0001), and *Ocln* (*F*_2,30_ = 24.11, *p* < 0.0001) levels were significantly decreased in both male and female TBI + saline groups compared with the Sham based on treatment. Moreover, *Inos*, *Nos3*, and *Ocln* levels were significantly decreased in the male TBI + DEX compared with the male Sham. *Ocln* expression was significantly decreased in the female TBI + saline compared with the female TBI + DEX. *Nos3* and *Ocln* levels were significantly decreased in male TBI + DEX compared with the female TBI + DEX (Figure 3A–C).

**FIGURE 3:**
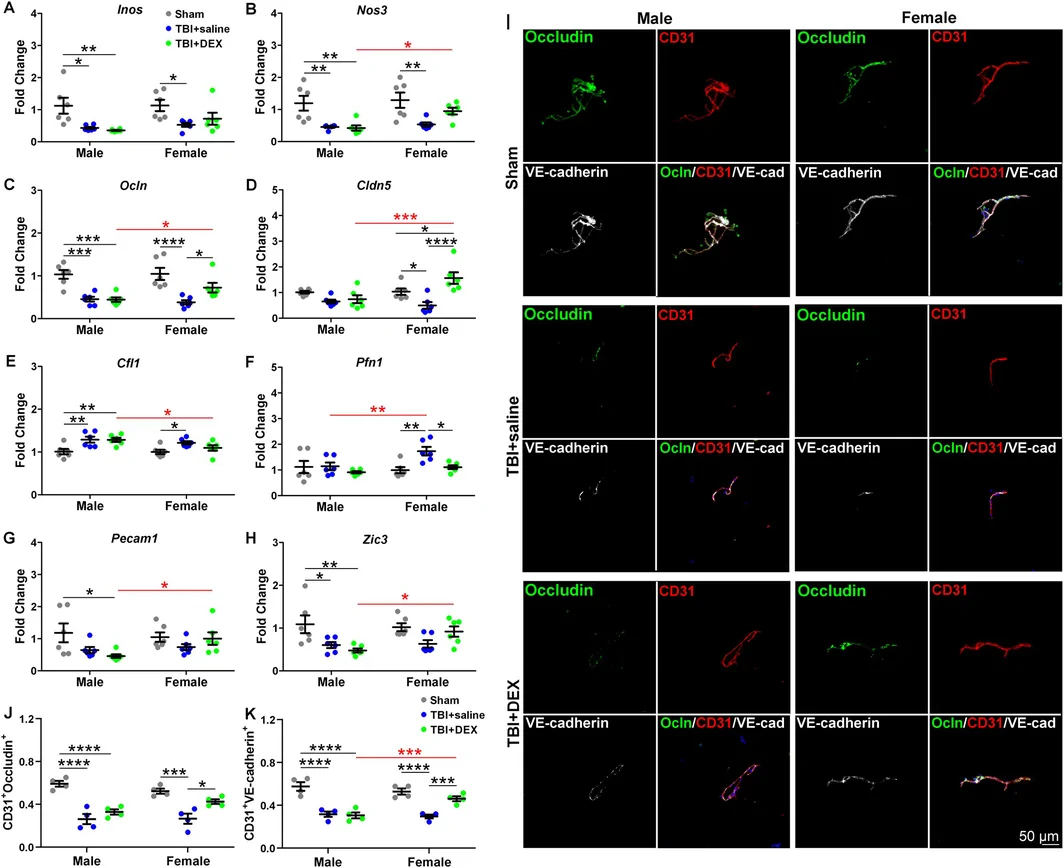
Effect of DEX on BMEC function. The mRNA expression of *Inos* (A), *Nos3* (B), *Ocln* (C), *Cldn5* (D), *Cfl1* (E), *Pfn1* (F), *Pecam1* (G), and *Zic3* (H) in Sham, TBI + saline, and TBI + DEX groups in both male and female mice (*n* = 6 per group). (I) Representative images of isolated blood vessels from male and female Sham, TBI + saline, and TBI + DEX groups showing immunostaining for occludin (green), VE‐cadherin (white), and the BMEC marker CD31 (red). Scale bar: 50 μm. (J) Co‐localization analysis of occludin protein expression with BMECs in isolated blood vessels (*n* = 4 per group). (K) Co‐localization analysis of VE‐cadherin protein expression with BMECs in isolated blood vessels (*n* = 4 per group). Statistical analysis was performed using two‐way ANOVA (sex [male, female], treatment [Sham, TBI + saline, TBI + DEX]) and Bonferroni's multiple‐comparison test. *p < 0.05; **p < 0.01; ***p < 0.001; ****p < 0.0001. *Cfl1*, cofilin‐1; *Cldn5*, claudin‐5; *Inos*, inducible nitric oxide synthase; *Nos3*, nitric oxide synthase 3; *Ocln*, occludin; *Pecam1*, platelet endothelial cell adhesion molecule 1; *Pfn1*, profilin‐1; *Zic3*, zic family member 3. Red annotations indicate sex‐related effects, whereas black annotations indicate treatment‐related effects.

*Cldn5* expression was significantly influenced by treatment (*F*_2,30_ = 9.574, *p* = 0.0006), sex (*F*_1,30_ = 4.192, *p* = 0.0495), and interaction (treatment × sex) (*F*_2,30_ = 7.045, *p* = 0.0031). In females, levels were lower in the TBI + saline group than in the Sham and TBI + DEX groups, while the female TBI + DEX group exhibited higher expression than both the female Sham and male TBI + DEX groups (Figure 3D). *Cfl1* also showed a significant treatment effect (*F*_2,30_ = 10.08, *p* = 0.0004), with elevated levels in both male and female TBI + saline groups relative to their respective Sham groups. Levels were also higher in male TBI + DEX animals than in male Sham and female TBI + DEX animals (Figure 3E). Similarly, *Pfn1* varied by treatment (*F*_2,30_ = 5.254, *p* = 0.0111), with greater expression in the female TBI + saline group than in the female Sham, female TBI + DEX, and male TBI + saline groups (Figure 3F). *Pecam1* was lower in male TBI + DEX animals than in male Sham and female TBI + DEX animals (*F*_2,30_ = 3.944, *p* = 0.0301) (Figure 3G). Finally, treatment significantly affected *Zic3* expression (*F*_2,30_ = 8.103, *p* = 0.0015). In males, levels were reduced in both TBI + saline and TBI + DEX groups relative to Sham, with the male TBI + DEX group also exhibiting lower expression than its female counterpart (Figure 3H).

Occludin protein levels in BMECs from isolated cerebral blood vessels were significantly affected by treatment (*F*_2,18_ = 38.29, *p* < 0.0001). VE‐cadherin protein levels were also significantly affected by treatment (*F*_2,18_ = 40.87, *p* < 0.0001) and by the treatment × sex interaction (*F*_2,18_ = 7.872, *p* = 0.0035). Compared with the male Sham, both the male TBI + saline and male TBI + DEX groups showed significantly reduced occludin and VE‐cadherin levels. In females, occludin and VE‐cadherin levels were significantly decreased in the TBI + saline group compared with both the female Sham and TBI + DEX groups. In addition, VE‐cadherin levels were significantly lower in the male TBI + DEX group than in the female TBI + DEX group (Figure 3I–K).

### 3.5 DEX Decreased Proinflammatory Responses in BMECs

To determine the effect of DEX on neuroinflammatory responses, the mRNA levels of *Tnf‐α*, *Il1b*, *Il6*, *Tgfb1*, *Tlr4*, *Nlrp3*, *Nfκb*, and *Icam1* were evaluated in BMECs of male and female mice. *Tnf‐α* expression was higher in both male and female TBI + saline groups than in the corresponding Sham and TBI + DEX groups, indicating a significant treatment effect (*F*_2,30_ = 18.78, *p* < 0.0001) (Figure 4C). A similar treatment effect was observed for *Il1b* (*F*_2,30_ = 24.14, *p* < 0.0001); expression was elevated in both TBI + saline groups relative to their respective Sham and TBI + DEX groups and was higher in females than in males following TBI + saline treatment (Figure 4D). *Il6* mRNA expression differed significantly by sex (*F*_1,30_ = 6.780, *p* = 0.0142) (Figure 4E), whereas no significant differences were detected in *Tgfb1* expression (Figure 4F). *Tlr4* expression also showed a significant treatment effect (*F*_2,30_ = 31.98, *p* < 0.0001), with elevated levels in male and female TBI + saline groups relative to the corresponding Sham and TBI + DEX groups. In addition, male TBI + DEX animals had lower *Tlr4* expression than male Sham animals (Figure 4G). *Nlrp3* expression showed a significant treatment effect (*F*_2,30_ = 27.72, *p* < 0.0001), with higher levels in both male and female TBI + saline groups than in the corresponding Sham and TBI + DEX groups (Figure 4H). Treatment also significantly affected *Nfκb* expression (*F*_2,30_ = 32.24, *p* < 0.0001). In males, expression was elevated following TBI + saline relative to Sham and TBI + DEX but was lower in TBI + DEX than in Sham. In females, expression was lower in TBI + DEX than in both Sham and TBI + saline groups (Figure 4I). Similarly, *Icam1* expression varied by treatment (*F*_2,30_ = 16.93, *p* < 0.0001). Male TBI + saline animals exhibited higher levels than their Sham and TBI + DEX counterparts, whereas female TBI + saline and TBI + DEX animals had higher levels than female Sham animals (Figure 4J).

**FIGURE 4:**
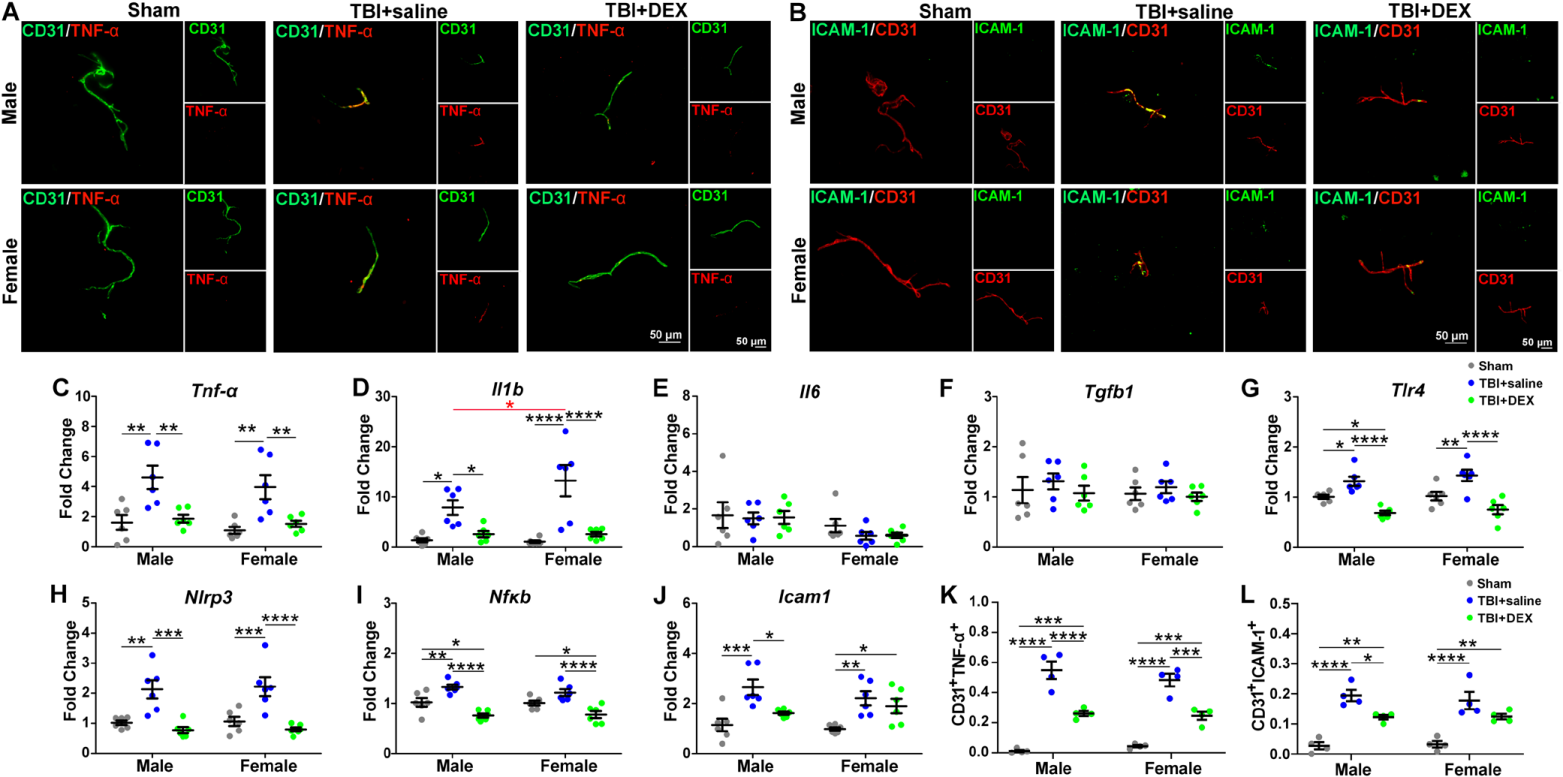
Expression of proinflammatory markers. (A) Representative immunohistochemical images displaying co‐localization of CD31 (a BMEC marker; green) and TNF‐α (a proinflammatory marker; red) in isolated blood vessels obtained from male and female mice in the Sham, TBI + saline, and TBI + DEX groups. Scale bar: 50 μm. (B) Representative immunohistochemical images displaying co‐localization of ICAM‐1 (green) and CD31 (red) in isolated blood vessels obtained from male and female Sham, TBI + saline, and TBI + DEX treatment groups. Scale bar: 50 μm. (C–J) The mRNA expression of *Tnf‐α* (C), *Il1b* (D), *Il6* (E), *Tgfb1* (F), *Tlr4* (G), *Nlrp3* (H), *Nfκb* (I), and *Icam1* (J) in the isolated BMECs from Sham, TBI + saline, and TBI + DEX groups in both male and female mice (*n* = 6 mice per group). (K) Quantitative analysis of the co‐localization between CD31 and TNF‐α was performed on isolated blood vessels from male and female mice across Sham, TBI + saline, and TBI + DEX treatment groups (*n* = 4 mice per group). (L) Quantitative analysis of the co‐localization between CD31 and ICAM‐1 was performed on isolated blood vessels from male and female Sham, TBI + saline, and TBI + DEX treatment groups (*n* = 4 mice per group). Statistical analysis was performed using two‐way ANOVA (sex [male, female], treatment [Sham, TBI + saline, TBI + DEX]) and Bonferroni's multiple‐comparison test. *p < 0.05; **p < 0.01; ***p < 0.001; ****p < 0.0001. *Icam‐1*, intercellular adhesion molecule 1; *Il‐1β*, interleukin‐1 beta; *Il‐6*, interleukin‐6; *Nfκb*, nuclear factor kappa‐light‐chain‐enhancer of activated B cells; *Nlrp3*, NOD‐, LRR‐, and pyrin domain‐containing protein 3; *Tgf‐β1*, transforming growth factor beta 1; *Tlr4*, toll‐like receptor 4; *Tnf‐α*, tumor necrosis factor‐alpha. Red annotations indicate sex‐related effects, whereas black annotations indicate treatment‐related effects.

Next, immunocytochemistry was performed on isolated blood vessels to assess TNF‐α and ICAM‐1 protein levels in BMECs. TNF‐α (*F*_2,18_ = 113.7, *p* < 0.0001) and ICAM‐1 (*F*_2,18_ = 47.19, *p* < 0.0001) levels were significantly affected by treatment, with increased levels observed in both male and female TBI + saline and TBI + DEX groups. TNF‐α levels were significantly higher in BMECs from both male and female TBI + saline groups compared with their respective TBI + DEX groups. In contrast, ICAM‐1 levels were significantly increased in the male TBI + saline group compared with the male TBI + DEX group (Figure 4A,B,K,L).

### 3.6 DEX Reduced ER Stress Responses in BMECs

To determine the effect of DEX on ER stress responses, the mRNA levels of *Ire1α* and *Perk* were evaluated in isolated BMECs from male and female mice. In males, *Ire1α* expression was significantly decreased in the TBI + DEX compared with the TBI + saline. In females, *Ire1α* expression was significantly decreased in the TBI + DEX compared with the Sham and TBI + saline based on treatment (*F*_2,30_ = 10.84, *p* = 0.0003) (Figure 5C). *Perk* mRNA expression was significantly decreased in the male TBI + DEX compared with the male TBI + saline based on treatment (*F*_2,30_ = 6.537, *p* = 0.0044) (Figure 5D).

**FIGURE 5:**
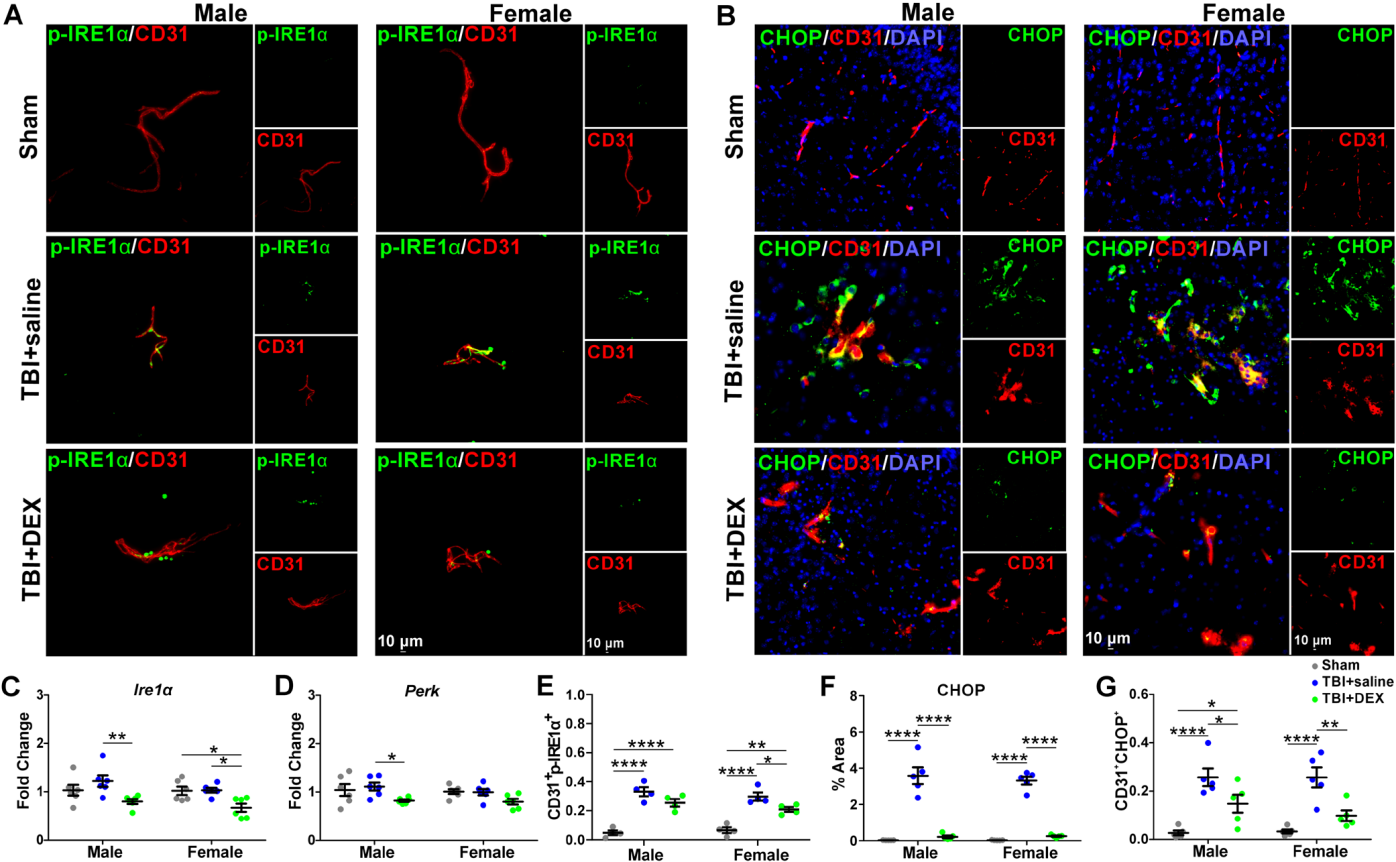
Effects of DEX on ER stress responses. (A) Representative immunohistochemical images displaying co‐localization of phosphorylated IRE1α (green) and CD31 (red) in isolated blood vessels obtained from male and female Sham, TBI + saline, and TBI + DEX groups. Scale bar: 10 μm. (B) Representative images of CHOP expression. CHOP (green), CD31 (red; a BMEC marker), and DAPI (blue; a nuclear marker). Scale bar: 10 μm. (C) mRNA expression of *Ire1α* in the isolated BMECs (*n* = 6 per experimental group). (D) mRNA expression of *Perk* in the isolated BMECs (*n* = 6 per experimental group). (E) Quantitative analysis of the co‐localization between CD31 and phosphorylated IRE1α was performed on isolated blood vessels from male and female Sham, TBI + saline, and TBI + DEX treatment groups (*n* = 4 mice per group). (F) CHOP protein expression was significantly higher in brain tissues from the TBI + saline groups (*n* = 5 per experimental group). (G) Co‐localization analysis of CHOP and BMECs (CD31‐positive cells) in brain tissues across all experimental groups (*n* = 5 per experimental group). Statistical analysis was performed using two‐way ANOVA (sex [male, female], treatment [Sham, TBI + saline, TBI + DEX]), and Bonferroni's multiple‐comparison test. *p < 0.05; **p < 0.01; ***p < 0.001; ****p < 0.0001.

Immunocytochemistry was performed on isolated blood vessels to assess phosphorylated IRE1α protein levels in BMECs. Phosphorylated IRE1α levels were significantly affected by treatment (*F*_2,18_ = 61.64, *p* < 0.0001), with increased levels observed in both male and female TBI + saline and TBI + DEX groups. Additionally, phosphorylated IRE1α levels were significantly higher in the female TBI + saline group compared with the female TBI + DEX group (Figure 5A,E).

Next, we analyzed the protein levels of CHOP in male and female mice using immunohistochemistry. CHOP levels were significantly increased in both male and female TBI + saline groups compared with the Sham and TBI + DEX based on treatment (*F*_2,24_ = 163.82, *p* < 0.0001) (Figure 5B,F). We further evaluated CHOP expression in BMECs by employing immunostaining and co‐localization analysis in brain tissue samples from both male and female mice. In males, CD31^+^/CHOP^+^ double‐positive cells were significantly increased in the TBI + saline compared with the Sham and TBI + DEX. Moreover, CD31^+^/CHOP^+^ double‐positive cells were significantly increased in the TBI + DEX compared with the Sham. In females, CD31^+^/CHOP^+^ double‐positive cells were significantly increased in the TBI + saline compared with the Sham and TBI + DEX based on treatment (*F*_2,24_ = 30.51, *p* < 0.0001) (Figure 5G).

### 3.7 DEX Decreased Ferroptosis‐Related Oxidative Stress in BMECs

To determine the effect of DEX on ferroptosis, the mRNA levels of *Gpx4*, *Ptgs2*, *Hmox1*, and *Tfrc* were evaluated in BMECs isolated from male and female mice. Treatment significantly affected *Gpx4* expression (*F*_2,30_ = 12.35, *p* = 0.0001). In males, expression was higher in the TBI + saline group than in the TBI + DEX group, whereas in females, it was higher than in both the Sham and TBI + DEX groups (Figure 6C). *Ptgs2* (*F*_2,30_ = 22.06, *p* < 0.0001) and *Hmox1* (*F*_2,30_ = 52.06, *p* < 0.0001) levels were significantly increased in both male and female TBI + saline groups compared with the Sham and TBI + DEX groups based on treatment (Figure 6D,E). *Tfrc* expression was significantly decreased in both male and female TBI + saline groups compared with the Sham based on treatment (*F*_2,30_ = 14.73, *p* < 0.0001) (Figure 6F).

**FIGURE 6:**
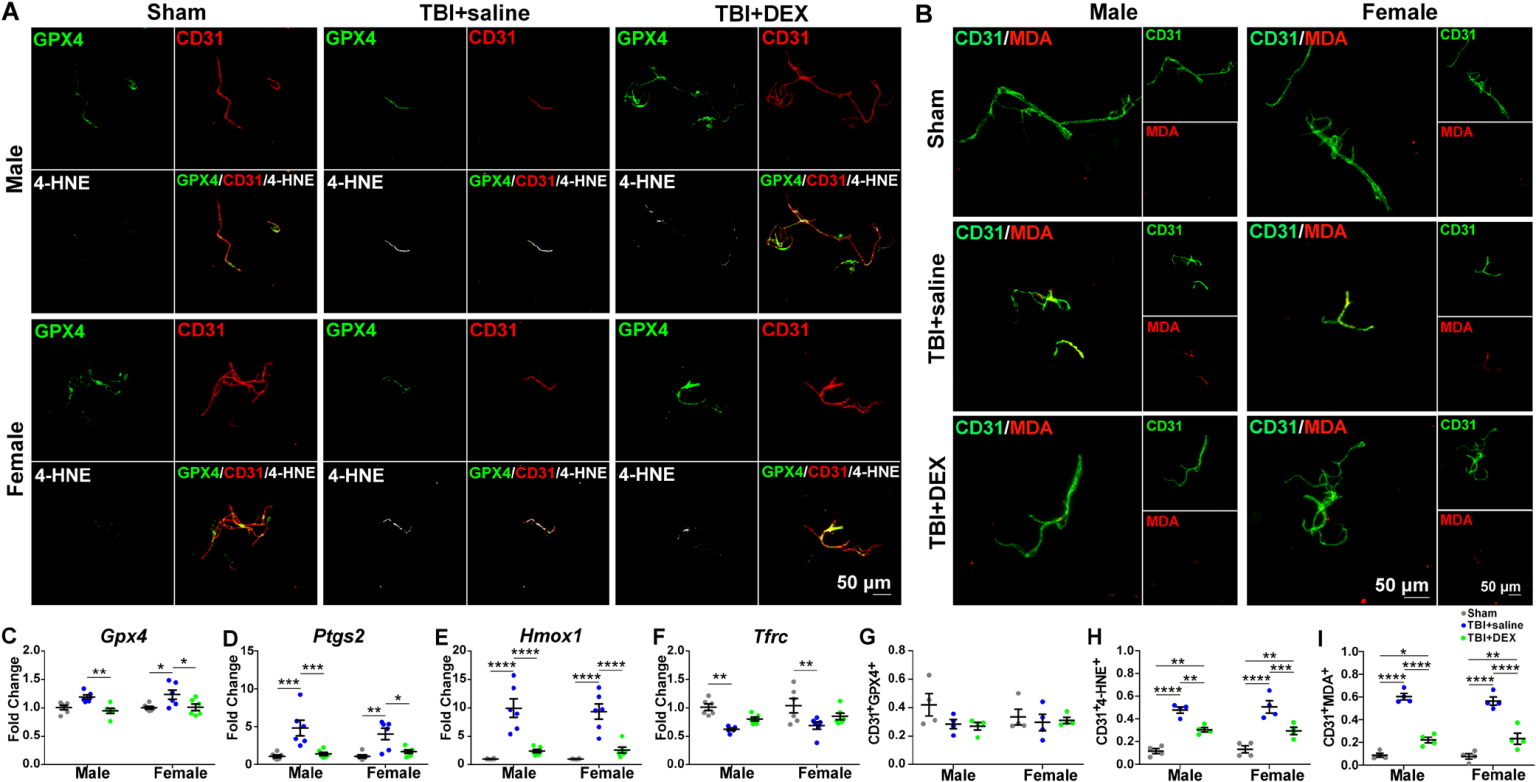
Effect of DEX on ferroptosis. (A) Representative immunohistochemical images displaying co‐localization of GPX4 (green), 4‐HNE (white), and CD31 (red) in isolated blood vessels obtained from male and female Sham, TBI + saline, and TBI + DEX groups. Scale bar: 50 μm. (B) Representative immunohistochemical images displaying co‐localization of CD31 (green) and MDA (red) in isolated blood vessels obtained from male and female mice in the Sham, TBI + saline, and TBI + DEX groups. Scale bar: 50 μm. (C–F) The mRNA expression of *Gpx4* (C), *Ptgs2* (D), *Hmox1* (E), and *Tfrc* (F) in isolated BMECs from Sham, TBI + saline, and TBI + DEX groups in both male and female mice (*n* = 6 per experimental group). (G–I) Quantitative analyses of co‐localization between CD31 and GPX4 (G), 4‐HNE (H), or MDA (I) were performed on isolated blood vessels from male and female mice across Sham, TBI + saline, and TBI + DEX treatment groups (*n* = 4 per experimental group). Statistical analysis was performed using two‐way ANOVA (sex [male, female], treatment [Sham, TBI + saline, TBI + DEX]), and Bonferroni's multiple‐comparison test. *p < 0.05; **p < 0.01; ***p < 0.001; ****p < 0.0001.

Immunocytochemistry was performed on isolated blood vessels to assess GPX4 protein expression in BMECs; no significant differences were observed among groups (Figure 6A,G). 4‐HNE (4‐hydroxynonenal) and MDA (malondialdehyde) are markers of lipid peroxidation associated with ferroptosis [40]. 4‐HNE (*F*_2,18_ = 63.08, *p* < 0.0001) and MDA (*F*_2,18_ = 130.2, *p* < 0.0001) levels were significantly increased in BMECs in the isolated cerebral microvessels from both male and female TBI + saline groups compared with the Sham and TBI + DEX groups based on treatment. Moreover, MDA levels were significantly increased in BMECs of both male and female TBI + DEX groups compared with the Sham (Figure 6A,B,H,I).

## 4 Discussion

BMEC dysfunction increases BBB permeability and exacerbates secondary injury processes, such as inflammation and oxidative stress, thereby contributing to TBI progression. Restoring BMEC function aids recovery. DEX, a selective α2‐adrenergic agonist, is used to manage agitation in patients with TBI [41] and has shown benefits by reducing neuroinflammation, oxidative stress, excitotoxicity, and apoptosis [17, 18, 19]. Few studies have examined DEX's effects on BMECs during the acute phase after TBI in immature brains. Our study demonstrated that, in juvenile mice, DEX improved muscle strength and sensorimotor coordination, partially reduced BBB leakage, and attenuated proinflammatory responses in BMECs. Notably, TBI and DEX treatment both induced sex‐specific changes in BMEC inflammation, ER stress, and tight junction marker expression.

Our previous studies have demonstrated that TBI can cause sex‐ and cell‐type‐specific changes in neurons, microglia, and astrocytes [26, 27, 28, 29]. In this study, we found that BMECs from female TBI + saline mice had higher mRNA levels of *Il1b* and *Pfn1* than BMECs from males. IL‐1β can disrupt the BBB and promote cerebral edema [42]. Profilin regulates actin dynamics, and its loss in endothelial cells increases inflammation [43]. However, the impact of these sex‐specific changes requires further investigation.

Notably, responses to DEX also differed significantly between males and females. Compared with TBI + saline mice, DEX treatment decreased *Icam1* and *Perk* mRNA expression only in males, whereas it increased JAM‐A, occludin, and VE‐cadherin protein expression only in females. ICAM‐1 expression is normally low in BMECs but increases during inflammation [44]. PERK promotes both adaptive and proapoptotic signaling in response to ER stress [45], but its overactivation can impair neurons [46]. JAM‐A, occludin, and VE‐cadherin are vital for BBB integrity [47]. Thus, these transcriptional patterns suggest that DEX may promote cell survival in males by lowering *Icam1* and *Perk* mRNA expression. In females, DEX may enhance BBB integrity by increasing the expression of tight junction and adherens junction markers.

Moreover, mRNA levels of *Nos3*, *Cldn5*, *Pecam1*, and *Zic3* were significantly lower in the male TBI + DEX group than in the female TBI + DEX group. Endothelial nitric oxide synthase (eNOS; encoded by *Nos3*) maintains cerebral blood flow [48], claudin‐5 (encoded by *Cldn5*) supports tight junction function [49], PECAM‐1 aids angiogenesis, vascular integrity, and leukocyte migration [50, 51], and ZIC3 promotes endothelial differentiation and BBB formation [52]. The reduced expression levels of these genes in males suggest that DEX may be less effective at restoring or preserving BMEC function after TBI in males than in females. This interpretation is consistent with previous evidence indicating that females exhibit greater resilience to acute BBB disruption following mild blast TBI [53]. Nevertheless, these sex‐dependent changes should be interpreted as molecular differences that may reflect differential regulation of BBB‐ or endothelial‐related pathways. They do not provide definitive evidence of sex‐specific functional impairment or treatment failure. Although the reduced molecular response to DEX in males suggests sex‐dependent effects, the functional efficacy of DEX in both sexes remains to be determined using additional outcome measures and longer follow‐up periods. Protein‐level validation is also needed to confirm whether these transcriptional changes translate into altered protein expression and functional outcomes. Moreover, alterations in BMEC markers, inflammatory gene expression, or oxidative stress‐related pathways do not necessarily imply a reduction in lesion burden, preservation of neuronal populations, or attenuation of neurodegenerative processes.

Sex differences in DEX responses may arise from several factors. In juvenile mice, circulating testosterone, estradiol, and progesterone levels are generally low and stable in both sexes, consistent with incomplete maturation of the hypothalamic–pituitary‐gonadal axis before puberty [54]. Therefore, sex‐dependent effects in juvenile models may not be driven solely by current hormone levels but also by the organizational effects of perinatal hormones. For example, the perinatal testosterone surge in males contributes to sex differences in hippocampal neurogenesis, synaptic development, and cognition [55]. In addition, androgen receptor expression in the developing cortex and hippocampus varies by age and sex [55]. These developmental programs may influence neuroimmune tone, vascular function, and repair after TBI, thereby modifying DEX responsiveness.

The differential molecular responses observed between males and females may be partly attributable to sex‐dependent regulation of α2‐adrenergic receptors following TBI. Following TBI, *Adra2a* and *Adra2b* mRNA levels were significantly reduced in females but not in males. These receptors regulate norepinephrine release, neuronal excitability, inflammation, and cellular stress signaling [56]. Sex hormones can also affect α2‐adrenergic receptor density, regional distribution, ligand affinity, and downstream signaling in the brain [57]. Thus, males and females may differ in receptor expression, coupling, or downstream signaling after brain injury [58]. However, receptor abundance was assessed only by immunofluorescence; ligand binding, receptor coupling, and functional downstream signaling were not directly measured. Future studies should compare α2‐adrenergic receptor expression and activity in male and female brains after TBI. Selective receptor antagonists and genetic approaches could also determine whether this pathway contributes directly to the sex‐specific response to DEX.

Other mechanisms may contribute to these sex‐specific effects. Sex‐related transcriptomic and functional differences in endothelial cells [59, 60], together with differences in the regulation of BBB pathways [61], may affect DEX penetration, vascular protection, and therapeutic efficacy after TBI. DEX has also shown sex‐specific behavioral effects, including greater anxiolytic efficacy in females [62]. In addition, sex differences in drug metabolism and pharmacokinetics may alter DEX exposure in brain microvascular endothelial cells and other target cells [63]. Collectively, these developmental, receptor‐mediated, vascular, behavioral, and pharmacokinetic factors may contribute to the sex‐specific DEX responses observed in this study (Figure 7).

**FIGURE 7:**
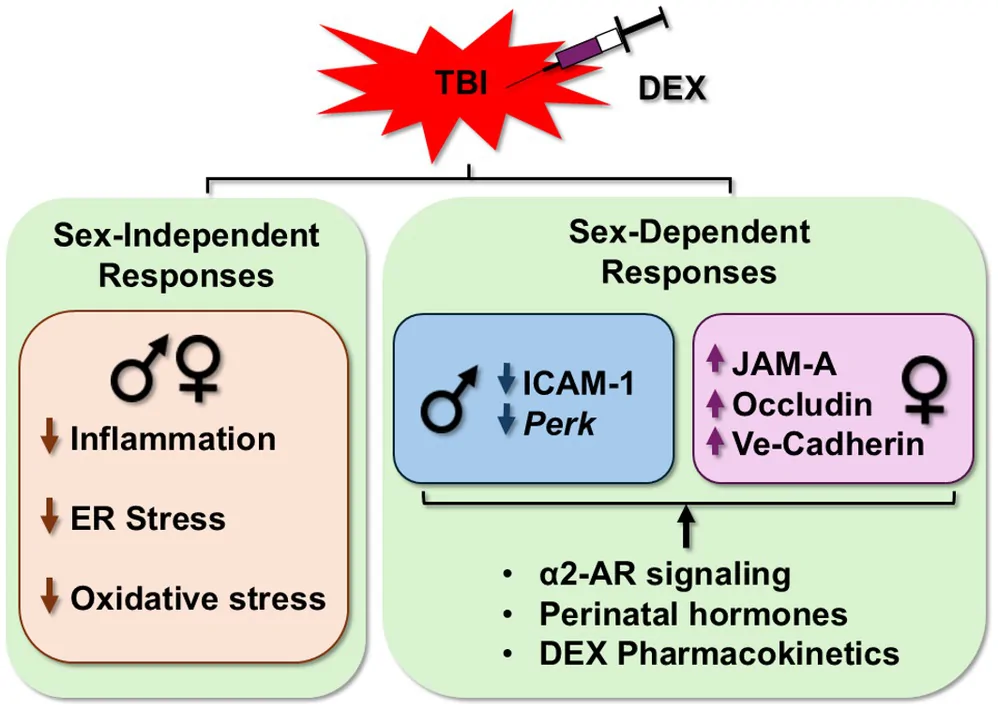
Schematic summary of the proposed sex‐independent and sex‐dependent mechanisms underlying DEX effects on BMECs.

Although this study provides important insights into sex‐specific responses to DEX in BMECs after TBI, it has several limitations. Several key markers, including eNOS, claudin‐5, ZIC3, TLR4, NLRP3, and PERK, were assessed exclusively at the transcript level. Therefore, the findings indicate transcriptional regulation but do not confirm changes in protein abundance or pathway activity. Transcript levels may not accurately reflect protein expression, posttranslational modifications, or functional signaling. Protein‐level measurements and functional assays are needed to strengthen the mechanistic interpretation. However, protein analysis of BMECs isolated from animals with TBI is limited by the small number of cells obtained from each sample. Conventional protein assays would require pooling many samples, which would increase animal use and reduce experimental feasibility. Future studies will use low‐input or ultrasensitive protein‐quantification methods and complementary functional assays. These approaches will help validate the identified targets while minimizing animal use.

Although DEX‐mediated protection may be associated, in part, with sex‐specific changes in α2‐adrenergic receptor transcripts, this study did not establish a causal relationship between α2‐AR signaling and neurovascular protection. DEX can also act on multiple cell types, including neurons and glial cells [30]. Therefore, the observed reductions in BBB leakage and injury‐related signaling may not result exclusively from α2‐AR activity in BMECs. Future studies will use selective α2‐AR antagonists and endothelial‐specific α2‐AR loss‐of‐function models to determine the role of this pathway. These studies will examine whether inhibition of α2‐AR signaling reduces the effects of DEX on Evans blue extravasation, lipid peroxidation, neuroinflammation, and BBB dysfunction after TBI. These experiments will clarify the causal contribution of α2‐AR signaling to the sex‐specific protective effects of DEX.

This study focused on the acute phase after injury and therefore could not determine whether the observed molecular changes provide sustained BBB protection or long‐term functional recovery. The 6‐h time point was selected to examine early processes associated with BBB disruption and secondary injury, including inflammation, oxidative stress, and changes in BBB‐related markers. However, acute molecular improvements may not indicate lasting neurovascular protection or behavioral benefits. Behavioral outcomes measured at 6 h may also have been affected by residual anesthesia and early injury‐related physiological changes. These factors may limit the interpretation of functional outcomes. Future studies should assess BBB permeability, tight junction integrity, neuroinflammation, and behavioral function at later time points. Such longitudinal analyses will determine whether the effects of DEX persist and contribute to long‐term tissue repair and functional recovery. Addressing these limitations will improve our understanding of the relationships among sex, ferroptosis, inflammation, and therapeutic responses after TBI. From a translational perspective, these findings should be interpreted with caution. Although DEX is currently used in pediatric clinical care, the dosing regimen employed in this murine study cannot be directly extrapolated to children because of species‐specific differences in pharmacokinetics, metabolism, route of administration, injury severity, and developmental physiology. The present findings suggest that DEX modulates acute BBB‐associated and molecular responses following juvenile TBI, potentially in a sex‐dependent manner. However, these data are insufficient to support sex‐specific clinical treatment recommendations. Translation is further constrained by the acute experimental time frame and the substantial heterogeneity of human pediatric TBI. Accordingly, additional dose–response, pharmacokinetic, longitudinal, and clinically aligned studies are required to determine the therapeutic relevance of DEX in pediatric TBI. Defining a clinically realistic therapeutic window will be essential before broader translational application can be considered.

## 5 Conclusions

TBI‐induced acute ER stress, inflammation, and ferroptosis‐related oxidative stress in BMECs, with several responses differing by sex. DEX produced both sex‐independent and sex‐dependent effects. It reduced markers of inflammation and ferroptosis‐related oxidative stress in both sexes but differentially affected ER stress and BBB‐related markers in males and females. These findings identify potential sex‐dependent molecular responses to DEX in BMECs of the developing brain and provide a basis for further investigation of its therapeutic relevance to pediatric TBI. Long‐term studies are required to determine the persistence and translational relevance of DEX‐mediated effects following TBI. Moreover, the association between α2‐adrenergic receptor signaling and the observed effects requires validation using pharmacological or genetic approaches.

## Author Contributions

Y.M., T.A., Z.N., S.M., and I.T. performed the experiments and contributed to writing the original draft. J.F. performed the experiments and contributed to visualization, verification, resource provision, writing the original draft, reviewing and editing the manuscript, and funding acquisition. Z.Z. conceptualized and designed the study and contributed to the investigation, formal analysis, visualization, verification, resource provision, writing the original draft, reviewing and editing the manuscript, supervision, project administration, and funding acquisition. All authors have seen and approved the final version of the manuscript being submitted.

## Funding

Research reported in this publication was supported by the National Institute of Neurological Disorders and Stroke of the National Institutes of Health under Award No. R15NS139138 to Zhi Zhang and Jie Fan. Additional support was provided to Zhi Zhang through a Start‐up Grant from the Department of Natural Sciences, College of Arts, Sciences, and Letters, University of Michigan–Dearborn, Grant No. U062955. The authors also acknowledge support from the EXP+ Student Independent Research Grant, University of Michigan–Dearborn.

## Ethics Statement

All experiments were conducted in accordance with the Guide for the Care and Use of Laboratory Animals, 8th edition (National Research Council, National Academies Press, 2011), and complied with the ARRIVE (Animal Research: Reporting of In Vivo Experiments) guidelines. Experimental procedures were approved by the Institutional Animal Care and Use Committee (IACUC) of the University of Michigan (Protocol number PRO00012565).

## Consent

The authors have nothing to report.

## Conflicts of Interest

The authors declare no conflicts of interest.

## Supporting information


**Data S1:** Materials and methods.

**Figure S1:** Complete datasets for immunohistochemical co‐localization analyses. Statistical analyses were conducted using two‐way ANOVA with sex (male, female) and treatment (Sham, TBI + saline, TBI + DEX) as factors, followed by Bonferroni's multiple comparisons test. Statistical significance is denoted as follows: *p < 0.05; **p < 0.01; ***p < 0.001; ****p < 0.0001. Red annotations indicate significant sex‐related effects, whereas black annotations indicate significant treatment‐related effects.

## Data Availability

The datasets generated and analyzed during this study are available from the corresponding author upon reasonable request.
