# Supplementary material for: Sex‐Specific Effects of Dexmedetomidine on Brain Microvascular Endothelial Cell Function After Traumatic Brain Injury in Juvenile Mice
Source: CNS Neurosci Ther. 2026 Oct 12;32(10):e71204. doi: 10.1002/cns.71204 (PMC13657077; doi:10.1002/cns.71204)
Supplement: Supplementary file 1 [file CNS-32-e71204-s001.docx]

**Sex-specific effects of dexmedetomidine on brain microvascular endothelial cell function after traumatic brain injury in juvenile mice**

Yara Mashal, Jie Fan, Tia Atoui, Zahrah Naseer, Sara Moughn, Iman Tawil, Zhi Zhang*****

Department of Natural Sciences, College of Arts, Sciences, and Letters, University of Michigan-Dearborn, Dearborn, MI

**2. Materials and methods**

**2.1. Animals**

Male and female C57BL/6 breeder mice, aged 2–3 months, were obtained from The Jackson Laboratory (Bar Harbor, ME, USA) and maintained for in-house breeding. Offspring generated from these breeding pairs were used in the subsequent experimental procedures. All the pups were delivered naturally and remained with their mother after birth until weaning. All animals were housed under ambient conditions (20-22°C, 40-60% relative humidity, and a 12-h light/dark cycle) with free access to food and water. Multiple precautions, including adequate habituation, gentle handling, minimization of procedure duration, and the use of humane endpoints, were taken throughout the study to minimize pain and stress associated with experimentation. All experiments were conducted in accordance with the Guide for the Care and Use of Laboratory Animals, 8th edition (National Research Council, National Academies Press, 2011), and complied with the ARRIVE (Animal Research: Reporting of In Vivo Experiments) guidelines. The pre-specified interim analysis was performed in line with the principles of replacement, reduction, and refinement to minimize unnecessary use of experimental animals. Experimental procedures were approved by the Institutional Animal Care and Use Committee (IACUC) of the University of Michigan (Protocol number PRO00012565).

**2.2. Estimation of animal numbers**

Statistical power and sample size calculations were performed using “PS: power and sample size calculation” software (version 3.1.6; Department of Biostatistics, Vanderbilt University, USA). Power calculations were performed with a significance level (α) of 0.05 (two-tailed), power of the study (1 - β) of 80% and variances (δ, σ, and m) estimated from our preliminary data. Based on these parameters, the required sample sizes were estimated to be 5–10 animals per group for behavioral assessments and 4–6 animals per group for immunohistochemical and quantitative real-time PCR (qPCR) analyses. In addition, the pre-specified interim analysis was performed in accordance with the “replacement, reduction, and refinement” rules to avoid unnecessary use of experimental animals.

**2.3. Impact acceleration model of TBI**

On postnatal day 20-21 (P20-21), male (M) and female (F) animals (n = 128, 64M/64F) from the same litter were randomized into Sham (n = 44, 22M/22F), TBI+saline (n = 44, 22M/22F) and TBI+DEX (n = 40, 20M/20F) groups using a random number generator. Randomization was stratified by sex. The TBI animals underwent injury procedures as previously described (Hamood, Abdullah et al. 2022, Faulkner, Rizk et al. 2023, Davila-Valencia, Saad et al. 2024, Dhull, Zhang et al. 2024, Ghannam, Hahn et al. 2024, Fan, Tasevski et al. 2025). In brief, anesthesia was induced with 4% Fluriso (Isoflurane, USP) (Cat# 502017, VetOne, MWI Animal Health, ID, USA) via inhalation and tail and/or paw pinches were used to ensure the animal was fully sedated. After fully anesthetized, the animal was placed chest-down on a platform with a trapdoor that supported the body weight of a mouse (~7-10 g body weight) with little to no resistance or restraint upon impact. The animal’s head was directly in the path of a falling weight. A weight (30g) was held 1.0 meters above the platform and secured by a pin. The lab personnel pulled the pin, allowing the weight to fall vertically through the guide tube to strike the animal on the head in the midline around 2 mm behind the eyes. The animal rapidly underwent a 180° rotation, falling through the trapdoor and landing in a supine position on a cushion. The animal was removed immediately from the apparatus and placed in a clean warm cage. Sham animals were anesthetized with 4% isoflurane without TBI impact. All animals were closely monitored postoperatively with health surveillance recordings, as per IACUC guidelines.

**2.4. Enzymatic isolation of primary BMECs and ferroptotic lipid peroxidation evaluation**

Animals [Sham = 4 (2M/2F); TBI = 4 (2M/2F)] were euthanized by inhalational administration of 5% Fluriso (Isoflurane, USP; Cat# 502017, VetOne, MWI Animal Health, ID, USA) until the onset of respiratory arrest, after which death was confirmed by decapitation. Brains were harvested and rinsed in DMEM (Cat# D5030; MilliporeSigma, MA, USA) on ice. Meninges were removed and the cortical injury region (approximately between bregma +2 mm and bregma -1 mm) in the TBI mice, and the matching area in the sham mice were micro-dissected as previously described (Hamood, Abdullah et al. 2022). Primary BMECs were isolated according to a previously published protocol (Ruck, Bittner et al. 2014). In brief, brain tissues were minced and incubated in collagenase CLS2 (final concentration of 0.5 mg/mL) (Cat# LS004176; Worthington Biochemical Corporation; NJ, USA) and DNase (final concentration of 20 µg/mL) (Cat# D4513; MilliporeSigma, MA, USA) in DMEM for 1 h at 37 °C on an orbital shaker at 180 rpm. Cells were centrifuged at 1,000 x g for 10 min at 4 °C. After myelin removal, cells were resuspended in BSA (Cat# A9418; MilliporeSigma, MA, USA)-DMEM (20% w/v) and centrifuged at 1,000 x g for 20 min at 4 °C. After myelin removal, cells were resuspended and incubated in collagenase/dispase (final concentration of 1 mg/mL; Cat#10269638001; Roche, CA, USA)/DNase (final concentration of 20 µg/ml) for 1 h at 37 °C on an orbital shaker at 180 rpm. Cell pellets were collected after centrifuging at 1,000 x g for 10 min at 4 °C and were resuspended in DMEM. The cell suspension was carefully added onto a Percoll gradient and centrifuged at 700 x g for 10 min at 4 °C. BMECs were collected from the interphase, washed in DMEM and centrifuged at 1,000 x g for 10 min at 4 °C. Cells were cultured in endothelial cell media [80% DMEM medium, 20% FBS (Cat# F4135, MilliporeSigma, MA, USA), 10 ng/mL bFGF (Cat# 100-18B; Thermo Fisher Scientific/Peprotech, NJ, USA), 15 U/mL heparin (Cat# H3393; MilliporeSigma, MA, USA) and 4 µg/mL puromycin (for first 2 days only; Cat# P8833; MilliporeSigma, MA, USA)].

The purity of cells was evaluated following our previously published protocol and the cell purity was calculated as the following: Cell purity = the number of CD31 positive cells/total number of cells x 100% (Ghannam, Hahn et al. 2024, Fan, Tasevski et al. 2025). To investigate the impact of TBI on ferroptosis, BMECs (320 cell/mm^2^) were plated and cultured in the micro-8-well slide (Cat# 80826; ibidi USA, Inc. WI, USA) in culture media. Cells were then incubated with BioTracker 633 Red Mitochondria Dye (Cat# SCT137; final concentration of 100 nM; MilliporeSigma, MA, USA) and BioTracker™ Mitochondrial Lipid Peroxide Live Cell Ferroptosis Indicator (Cat# SCT261; final concentration of 100 nM; MilliporeSigma, MA, USA) in culture media for 30 min before imaging. Images were acquired using a Nikon Plan Apo objective (20X/0.9) through the Nikon Eclipse TS2R fluorescent microscope (Nikon, NY, USA).

**2.5. Drug administration**

Dexdomitor (dexmedetomidine hydrochloride, concentration of 0.5 mg/mL) was purchased from MWI Animal Health (Fenton, MI, USA) and diluted in 0.9% sterile saline at 10 μg/mL concentration. Animals in the TBI+DEX group received intraperitoneal injection (IP) of DEX (25 µg/kg) at 15 min post-injury (Fan, Tasevski et al. 2025). The TBI+saline group received the same volume of saline at 15 min post-injury. The sham group did not receive any treatment.

**2.6. Body weight measurement and behavioral testing**

Studies have shown that DEX can be extensively distributed and rapidly eliminated with a distribution half-life of 6 minutes and a mean elimination half-life of 2-3 hours (Weerink, Struys et al. 2017). To avoid DEX interference, behavioral tests were performed at 6-hour (h) post-injury at which time the mice were fully recovered and resumed normal activities.

Mice from the Sham (n = 40, 20M/20F), TBI+saline (n = 40, 20M/20F) and TBI+DEX (n = 40, 20M/20F) groups underwent body weight measurement and behavioral testing before injury (baseline) and at 6-h post-injury. The personnel were blinded to experimental groups. All animals underwent the testing in the following orders: the body weight, grip strength and Rotarod. There was a 20-30 min break after each test, so the animal could rest and recover.

**2.6.1. Body weight**

Body weight was measured before injury (baseline) and at 6-h post-injury. The changes in the body weight were calculated as (body weight) _change_ = (body weight)_6h_ – (body weight)_baseline._

**2.6.2. Grip strength**

Muscular strength was evaluated with a grip strength test using a grip strength meter (BIOSEB, FL, USA) before injury and at 6-h post-injury (Ghannam, Hahn et al. 2024, Fan, Tasevski et al. 2025). In brief, the grip strength meter was positioned horizontally, and the animals were held by the tail and lowered towards the apparatus. The animals were allowed to grab the metal grid and were then pulled backwards in the horizontal plane. The force applied to the grid was recorded as the peak tension. Each animal underwent the grip strength test in three consecutive trials. The results were recorded and averaged for each animal. The change in the grip strength before and after injury was calculated as: (grip strength) _change_ = (grip strength) _6h_ – (grip strength) _baseline_

**2.6.3. Rotarod**

Sensorimotor coordination, endurance, and fatigue resistance were evaluated with a touchscreen five-station accelerating Panlab Rota Rod for mice (BIOSEB, FL, USA) before injury and at 6-h post-injury based on a published protocol (Zhang and van Praag 2015, Ghannam, Hahn et al. 2024, Fan, Tasevski et al. 2025). Each animal was situated on a stationary rod for 10 s, and the rod was then set in motion with an accelerating speed of 3–30 rpm. Each animal underwent three consecutive trials, and the maximal duration of each trial was 5 min. For each trial, if the animal fell before it reached the 5-min maximum test time, the trial ended at the time of the fall, and the latency to the fall was recorded. The latency to the first fall in three consecutive trials were recorded and averaged for each animal. The change in the latency to the first fall before and after injury was calculated as: (latency) _change_ = (latency) _6h_ – (latency) _baseline_

**2.7. Evans blue extravasation assay**

Evans blue extravasation assay was modified based on published protocols (Wang and Lai 2014, Assis-Nascimento, Tsenkina et al. 2018). Animals [Sham = 10 (5M/5F); TBI+saline = 10 (5M/5F); TBI+DEX = 10 (5M/5F)] received an intravenous injection of 10 mg/kg of 2% sterile Evans blue (Cat# A16774.18; Thermo Fisher Scientific, MA, USA) via tail vein after completion of behavioral tests. Animals were euthanized at 2-h post-Evans blue injection. Euthanasia was performed by administering 5% Fluriso (Isoflurane, USP) (Cat# 502017, VetOne, MWI Animal Health, ID, USA) via inhalation until cessation of respiration was observed, followed by transcardial perfusion with phosphate-buffered saline (PBS) to ensure death. Brains were removed and the cortical injury regions (approximately between bregma +2 mm and bregma -1 mm) in the TBI mice, and the matching area in the sham mice were micro-dissected. Brain tissues were weighed, homogenized in 50% solution of trichloroactic acid (Cat# T9159; Sigma-Aldrich, MA, USA) and centrifuged at 10,000 x g for 20 min. The supernatant absorbance was measured at 610 nm using a NanoDrop 2000 Spectrophotometer (Thermo Fisher Scientific, MA, USA), and trichloroactic acid was used as the blank. Evans blue absorbance was quantified according to Evans blue external standard curve (1-50 ng/mL) and converted into nanogram of dye extravasated per milligram of tissue. The Evans blue concentrations were then normalized based on the results for the sham animals and compared among treatment groups.

**2.8. Isolation of cerebral microvessels and immunohistochemistry**

Animals were euthanized after completion of behavioral tests [Sham = 8 (4M/4F); TBI+saline = 8 (4M/4F); TBI+DEX = 8 (4M/4F)], the cerebral microvessels were isolated based on a published protocol (Lee, Uchida et al. 2019). In brief, animals were euthanized by inhalational administration of 5% Fluriso (Isoflurane, USP; Cat# 502017, VetOne, MWI Animal Health, ID, USA) until the onset of respiratory arrest, after which death was confirmed by decapitation. Brains were harvested, and the meninges and meningeal vessels were removed. Brains were rinsed in MCDB 131 medium (no glutamine; Cat# 10372019, Thermo Fisher Scientific, MA, USA) and the cortical injury regions (approximately between bregma +2 mm and bregma -1 mm) in the TBI mice, and the matching area in the sham mice were micro-dissected as previously described (Hamood, Abdullah et al. 2022, Dhull, Zhang et al. 2024, Dar AI, Zhang Z et al. 2025). Brain tissues were homogenized in MCDB 131 medium using a loose-fit, 7-ml a Dounce tissue homogenizer (Thermo Fisher Scientific, MA, USA), and centrifuged at 2,000 xg for 5 min at 4 °C. The supernatant was discarded, the pellet was resuspended in 15% (wt/vol) dextran (Cat# 31390, Sigma-Aldrich, MA, USA)-DPBS (Cat# 14190136, Thermo Fisher Scientific, MA, USA), and centrifuge at 10,000 xg for 15 min at 4 °C. The supernatant was discarded, and the pellet was transferred to a 40-μm cell strainer (Cat#22-363-547; Fisher Scientific, MA, USA) and washed through with 9 mL of DPBS. The microvessels were fixed in 4% (wt/vol) PFA-DPBS for 15 min with gentle shaking. The microvessels were washed in 20 mL of 1% (wt/vol) BSA-DPBS, centrifuged at 2,000 x g for 10 min at 4 °C.

The microvessels were incubated with primary antibodies overnight at 4°C. The primary antibodies utilized were as follows: rat anti-CD31, an endothelial marker (1:250; Cat. No. MA1-40074; Thermo Fisher Scientific, MA, USA); goat anti-ADRA2A (1:50; Cat. No. NB100-2819; R&D Systems, Bio-Techne, MN, USA); rabbit anti-ADRA2B (1:50; Cat. No. 63-520; ProSci, CA, USA); rabbit anti-junctional adhesion molecule 1/JAM-A (1:250; Cat. No. ab125886; Abcam, MA, USA); rabbit anti-occludin (1:100; Cat. No. ab31721; Abcam, MA, USA); mouse anti-VE-cadherin (1:100; Cat. No. sc-9989; Santa Cruz Biotechnology, TX, USA); mouse anti-TNF-α (1:250; Cat. No. GTX35134; GeneTex, CA, USA); rabbit anti-ICAM-1 (1:50; Cat. No. 19-750; ProSci, CA, USA); rabbit anti-phospho-IRE1α (1:250; Cat. No. PA5-105424; Thermo Fisher Scientific, MA, USA); rabbit anti-GPX4 (1:50; Cat. No. 14-907; ProSci, CA, USA); mouse anti-4-HNE (1:250; Cat. No. MAB3249; R&D Systems, Bio-Techne, MN, USA); and mouse anti-malondialdehyde (MDA) (1:250; Cat. No. MA5-27560; Thermo Fisher Scientific, MA, USA). The microvessels were subsequently washed and incubated with fluorescent secondary antibodies (1:250; Life Technologies, MA, USA) for 2 h at room temperature. The microvessels were mounted with fluorescent mounting media with DAPI (Sigma-Aldrich, MO, USA). Images (40X magnification, 5 per animal) were acquired using the Nikon Eclipse TS2R fluorescent microscope (Nikon, NY, USA) with NIS-Elements AR 5.42.04 software package, and the camera settings were kept the same for all the experimental groups. All slides and images were coded, and the analysis was performed with personnel blinded to the experiments. The measurement data were averaged per animal and compared among groups.

**2.9. Isolation of primary BMECs using magnetic cell separation**

Following completion of behavioral assessments, animals [Sham = 12 (6M/6F); TBI+saline = 12 (6M/6F); TBI+DEX = 12 (6M/6F)] were euthanized by inhalational administration of 5% Fluriso (Isoflurane, USP; Cat# 502017, VetOne, MWI Animal Health, ID, USA) until respiratory arrest was achieved. Death was subsequently confirmed by decapitation. Brains were harvested and rinsed in HBSS solution on ice. Meninges were removed and the cortical injury regions (approximately between bregma +2 mm and bregma -1 mm) in the TBI mice, and the matching area in the sham mice were micro-dissected as previously described (Hamood, Abdullah et al. 2022). Brain tissues were homogenized in ice-cold HBSS with a Dounce tissue homogenizer (Thermo Fisher Scientific, MA, USA) and passed through 40 µm cell strainers (Cat#22-363-547; Fisher Scientific, MA, USA) to achieve a single‑cell suspension. Cell suspension was centrifuged at 300×g for 10 min at 4 °C, and supernatant was aspirated completely. Cells were re-suspended in ice-cold D-PBS (Cat# 14-040-117; Fisher Scientific, MA, USA)/ heat-inactivated fetal bovine serum (BSA; Cat# 16140071; Thermo Fisher Scientific, MA, USA) buffer (90 µL per 1×10⁷ total cells), 10 µL of mouse CD31 MicroBeads (Cat#130-097-418; Miltenyi Biotec, MD, USA) were added, mixed, and incubated at 4°C for 15 min in the dark. Cells were washed with ice-cold D-PBS/BSA buffer, centrifuged at 300×g for 10 min at 4°C, and the supernatant was aspirated completely. Cells were re-suspended in 500 μL of D-PBS/BSA buffer and separated using LS columns and a QuadroMACS™ Separator (Miltenyi Biotec, MD, USA). After washing, LS columns were removed from the separator and placed on 15 mL collection tubes. Magnetically labeled cells were flushed out and collected in ice-cold D-PBS/BSA buffers. Cells were centrifuged at 300×g for 10 min at 4°C, and cell pellets were collected.

**2.10. RNA isolation and quantitative real-time polymerase chain reaction (qPCR)**

Total RNA was extracted using TRI Reagent (Cat# T9424; Sigma-Aldrich, MO, USA), according to the manufacturer’s instructions. RNA samples were quantified using the Nanodrop ND-2000 Spectrophotometer (Thermo Fisher Scientific, MA, USA). Single-stranded complementary DNA (cDNA) was reverse transcribed from RNA using the High-Capacity cDNA Reverse Transcription Kit with RNase inhibitor (Cat# 4374966; Thermo Fisher Scientific, MA, USA). The qPCR was performed with iTaq(tm) Universal SYBR(R) Green Supermix (Cat# 1725125; Bio-Rad, CA, USA) and the CFX connect real-time PCR detection system (Bio-Rad, CA, USA). Amplification conditions included 30 s at 95°C, 40 cycles at 95°C for 5 s, and 60°C for 30 s. Primers were custom designed (Table 1) and ordered from Integrated DNA Technology (Coralville, IA, USA). The comparative threshold cycle (Ct) method was used to assess differential gene expressions. The sham group was the reference group, and glyceraldehyde 3-phosphate dehydrogenase (GAPDH) was the housekeeping gene. Gene expression levels for each sample were normalized to the expression level of GAPDH within a given sample (ΔCt); the differences between sham and TBI groups were used to determine the ΔΔCt. The 2-ΔΔCt gave the relative fold changes in gene expression. The results from sham and TBI groups were averaged and compared.

**2.11. Mouse brain tissue immunofluorescence staining and imaging**

Animals were euthanized after completion of behavioral tests [Sham = 10 (5M/5F); TBI+saline = 10 (5M/5F); TBI+DEX = 10 (5M/5F)]. Euthanasia was performed by administering 5% Fluriso (Isoflurane, USP) (Cat# 502017, VetOne, MWI Animal Health, ID, USA) via inhalation until cessation of respiration was observed, followed by transcardial perfusion with PBS to ensure death. Brains were removed, postfixed in 10% formalin for 48 h, and then cryoprotected in 30% sucrose (in PBS). Coronal sections (20 µm, 1:6 series) were prepared on a cryostat (Leica Microsystems, IL, USA). Brain sections were incubated with primary antibodies overnight at 4°C. Brain sections were subsequently washed and incubated with fluorescent secondary antibodies (1:250; Life Technologies, MA, USA) for 2 h at room temperature. The slides were dried, and cover-slipped with fluorescent mounting media with DAPI (Sigma-Aldrich, MO, USA). Images (40X magnification, 5 per animal) were randomly acquired from the cortical area of the injured brain regions (approximately between bregma +1 mm and bregma -0.5 mm) in TBI animals, or from the corresponding cortical regions in the Sham group using the Nikon Eclipse TS2R fluorescent microscope (Nikon, NY, USA) with NIS-Elements AR 5.42.04 software package, and the camera settings were kept the same for all the experimental groups. All slides and images were coded, and the analysis was performed with personnel blinded to the experiments. The measurement data were averaged per animal and compared among groups.

**2.12. Histology quantification**

The expression of CHOP was evaluated as the “percentage of area” using “particle analysis” function in Fiji ImageJ software (National Institutes of Health, NIH) as previously described (Zhang, Bassam et al. 2016, Zhang, Ishrat et al. 2020, Hamood, Abdullah et al. 2022, Faulkner, Rizk et al. 2023). In brief, the images were converted to 8-bit greyscale, and the threshold was manually adjusted, followed by application of the ‘analyze particles’ function. Finally, a visual check was performed utilizing the ‘merge channel” function in ImageJ.

Co-localization analyses of CD31^+^/CHOP^+^ in brain tissues, CD31^+^/ADRA2A^+^, CD31^+^/ADRA2B^+^, CD31^+^/JAM-A^+^, CD31^+^/ Occludin^+^, CD31^+^/VE-cadherin^+^, CD31^+^/TNF-α^+^, CD31^+^/ICAM1^+^, CD31^+^/ phospho-IRE1α^+^, CD31^+^/GPX4^+^, CD31^+^/4-HNE^+^ and CD31^+^/MDA^+^ in isolated cerebral micro-blood vessels were performed using “Coloc 2” function in Fiji ImageJ (National Institutes of Health, NIH) followed the manufacturer’s instructions (Ghannam, Hahn et al. 2024). In brief, images were split into separate channels and were converted to 8-bit. Background subtraction was performed using Fiji ImageJ following the manufacturer’s instructions. The co-localization was analyzed using Kendall's Tau Rank Correlation test, and Costes' randomization was set at 10. To ensure the accuracy of analysis, data were manually checked with co-localization analysis as described previously (Zhang, Bassam et al. 2016, Zhang, Ishrat et al. 2020, Hamood, Abdullah et al. 2022, Faulkner, Rizk et al. 2023, Fan, Tasevski et al. 2025).

**2.13. Statistical analysis**

Data were analyzed using GraphPad Prism (Version 10.4.1; CA, USA) and IBM SPSS Statistics [Version: 28.0.1.1 (15)]. All data were presented as mean ± SEM. D'Agostino and Pearson omnibus normality test or Kolmogorov–Smirnov test was used for normality measurement. Student's t-test (two-tailed) or Mann–Whitney U test (two-tailed) was used for two-group comparisons. Multiple comparisons were analyzed using one-way or two-way analysis of variance (ANOVA), followed by Bonferroni post hoc correction where appropriate. The Bonferroni method was used to adjust individual p values for multiple hypothesis testing and to control the family-wise error rate. In addition, the two-stage step-up procedure of Benjamini, Krieger, and Yekutieli was applied, where appropriate, to control the false discovery rate. Statistical significance was set at p<0.05 for all analyses.

**References**

Assis-Nascimento, P., Y. Tsenkina and D. J. Liebl (2018). "EphB3 signaling induces cortical endothelial cell death and disrupts the blood-brain barrier after traumatic brain injury." Cell Death Dis **9**(1): 7.

Dar AI, Zhang Z , Gopalakrishnan S, Sharma R, Pulukuri AJ, Rani A, Dhull A, Gonzalez JC, Atoui T, Mashal Y, Naseer Z, Calmi J and S. A. (2025). "Neuron-targeted 2-deoxyglucose-dendrimer-rosiglitazone nanotherapy mitigates neuroinflammation and cognitive deficits in pediatric traumatic brain injury." Bioengineering & Translational Medicine **2025;e70053**(2025;e70053): 1-26.

Davila-Valencia, I., M. Saad, G. Olthoff, M. Faulkner, M. Charara, A. Farnum, R. C. Dysko and Z. Zhang (2024). "Sex specific effects of buprenorphine on adult hippocampal neurogenesis and behavioral outcomes during the acute phase after pediatric traumatic brain injury in mice." Neuropharmacology **245**: 109829.

Dhull, A., Z. Zhang, R. Sharma, A. I. Dar, A. Rani, J. Wei, S. Gopalakrishnan, A. Ghannam, V. Hahn, A. J. Pulukuri, S. Tasevski, S. Moughni, B. J. Wu and A. Sharma (2024). "Discovery of 2-deoxy glucose surfaced mixed layer dendrimer: a smart neuron targeted systemic drug delivery system for brain diseases." Theranostics **14**(8): 3221-3245.

Fan, J., S. Tasevski, Y. Mashal, T. Atoui, Z. Naseer and Z. Zhang (2025). "Sex- and cell type-specific effects of dexmedetomidine on ferroptosis in neurons and microglia following traumatic brain injury in juvenile mice." Mol Neurobiol **63**(1): 4.

Faulkner, M. B., M. Rizk, Z. Bazzi, R. C. Dysko and Z. Zhang (2023). "Sex-Specific Effects of Buprenorphine on Endoplasmic Reticulum Stress, Abnormal Protein Accumulation, and Cell Loss After Pediatric Mild Traumatic Brain Injury in Mice." Neurotrauma Rep **4**(1): 573-585.

Ghannam, A., V. Hahn, J. Fan, S. Tasevski, S. Moughni, G. Li and Z. Zhang (2024). "Sex-specific and cell-specific regulation of ER stress and neuroinflammation after traumatic brain injury in juvenile mice." Exp Neurol: 114806.

Hamood, Y., M. Abdullah, H. El Ghoul, N. Saad, R. C. Dysko and Z. Zhang (2022). "Sex specific effects of buprenorphine on behavior, astrocytic opioid receptor expression and neuroinflammation after pediatric traumatic brain injury in mice." Brain Behav Immun Health **22**: 100469.

Lee, Y. K., H. Uchida, H. Smith, A. Ito and T. Sanchez (2019). "The isolation and molecular characterization of cerebral microvessels." Nat Protoc **14**(11): 3059-3081.

Ruck, T., S. Bittner, L. Epping, A. M. Herrmann and S. G. Meuth (2014). "Isolation of primary murine brain microvascular endothelial cells." J Vis Exp(93): e52204.

Wang, H. L. and T. W. Lai (2014). "Optimization of Evans blue quantitation in limited rat tissue samples." Sci Rep **4**: 6588.

Weerink, M. A. S., M. Struys, L. N. Hannivoort, C. R. M. Barends, A. R. Absalom and P. Colin (2017). "Clinical Pharmacokinetics and Pharmacodynamics of Dexmedetomidine." Clin Pharmacokinet **56**(8): 893-913.

Zhang, Z., B. Bassam, A. G. Thomas, M. Williams, J. Liu, E. Nance, C. Rojas, B. S. Slusher and S. Kannan (2016). "Maternal inflammation leads to impaired glutamate homeostasis and up-regulation of glutamate carboxypeptidase II in activated microglia in the fetal/newborn rabbit brain." Neurobiol Dis **94**: 116-128.

Zhang, Z., S. Ishrat, M. O'Bryan, B. Klein, M. Saraswati, C. Robertson and S. Kannan (2020). "Pediatric Traumatic Brain Injury Causes Long-Term Deficits in Adult Hippocampal Neurogenesis and Cognition." J Neurotrauma **37**(14): 1656-1667.

Zhang, Z. and H. van Praag (2015). "Maternal immune activation differentially impacts mature and adult-born hippocampal neurons in male mice." Brain Behav Immun **45**: 60-70.
