# Supplementary material for: Sex‐Specific Effects of Dexmedetomidine on Brain Microvascular Endothelial Cell Function After Traumatic Brain Injury in Juvenile Mice
Source: CNS Neurosci Ther. 2026 Oct 12;32(10):e71204. doi: 10.1002/cns.71204 (PMC13657077; doi:10.1002/cns.71204)
Supplement: Supplementary file 2 [file CNS-32-e71204-s002.docx]

**Sex-specific effects of dexmedetomidine on brain microvascular endothelial cell function after traumatic brain injury in juvenile mice**

Yara Mashal, Jie Fan, Tia Atoui, Zahrah Naseer, Sara Moughn, Iman Tawil, Zhi Zhang*****

Department of Natural Sciences, College of Arts, Sciences, and Letters, University of Michigan-Dearborn, Dearborn, MI

**Supplemental Figure 1**

**Supplemental Figure 1. Complete datasets for immunohistochemical co-localization analyses.** Statistical analyses were conducted using two-way ANOVA with sex (male, female) and treatment (Sham, TBI + saline, TBI + DEX) as factors, followed by Bonferroni’s multiple comparisons test. Statistical significance is denoted as follows: *, p < 0.05; ***,* p < 0.01; ***, p < 0.001; ****, p < 0.0001. Red annotations indicate significant sex-related effects, whereas black annotations indicate significant treatment-related effects.
